# CDX1 and CDX2 suppress colon cancer stemness by inhibiting β-catenin-facilitated formation of Pol II–DSIF–PAF1C complex

**DOI:** 10.1038/s41419-025-07737-3

**Published:** 2025-05-21

**Authors:** Koji Aoki, Akari Nitta, Ayumi Igarashi

**Affiliations:** 1 Department of Pharmacology, Faculty of Medicine, University of Fukui, Fukui, Japan; 2 Life Science Support Center, University of Fukui, Fukui, Japan

**Keywords:** Cancer stem cells, Colon cancer

## Abstract

Homeobox transcription factors CDX1 and CDX2 (hereafter, CDX1/2) play key roles in determining the identity of intestinal epithelial cells and regulating their stem cell functions. However, the role of CDX1/2 in regulating colon cancer stemness and the underlying mechanisms are unclear. Here, we show that complete loss of Cdx1 or concurrent loss of Cdx1/2 increased the stemness and malignancy of intestinal tumors. Consistently, CDX1/2 reduced the expression of cancer stemness-related genes, including *LGR5*. CDX1/2 bound to the downstream region of the *LGR5* transcription start site (TSS), a region where β-catenin also binds. Despite increased H3 acetylation and an open chromatin structure, CDX1/2 reduced the occupancy of DRB sensitivity-inducing factor (DSIF), RNA polymerase II-associated factor 1 (PAF1), and RNA polymerase II (Pol II) complexes around the *LGR5* TSS. Through their homeodomains, CDX1/2 inhibited the β-catenin-facilitated formation of active Pol II complexes containing DSIF and PAF1 complexes by preventing the interaction between β-catenin and these complexes, in an additive manner. Our findings suggest that CDX1/2 cooperatively suppressed colonic tumorigenesis and cancer stemness by antagonizing β-catenin via the DSIF and PAF1 complexes. Additionally, DSIF and PAF1 complexes acted as transcriptional platforms that integrated and funneled both tumor-suppressive and oncogenic signals into the expression of genes that control colon cancer stemness.

## Introduction

The intestine-specific homeoproteins CDX1/2 play a central role in determining the identity of intestinal epithelial cells, promoting their development and differentiation, and maintaining their homeostasis [1–8]. Previous data showed that CDX2 levels were reduced in human colon cancer tissues [9]. Consistently, *Cdx2* mutation or reduced Cdx2 expression enhances *Apc* mutation- or carcinogen-induced colonic tumorigenesis through cell-autonomous and non-cell autonomous mechanisms [10–16]. However, the cooperative role of CDX1/2 in malignant progression remains unclear.

Mutations in *APC*, found in ~80% of colon cancer [17–19], enhance β-catenin stabilization and induce colonic tumorigenesis [20–22]. Hence, *Apc* mutant mice have been used to genetically model colonic tumorigenesis [23]. In mice, *Cdx1* is located on chromosome 18 (Chr 18), approximately 13 centimorgan from *Apc*. When *Apc*^+/−^ mice were crossed with *Cdx1*^+/−^ mice, the resulting *Apc*^+/−^*Cdx1*^+/−^ mice carried mutant *Apc* and *Cdx1* alleles on different Chr 18 homologs (trans-*Apc*^+/−^*Cdx1*^+/−^). In *Apc*^+/−^ mice, intestinal tumorigenesis was initiated by the loss of Chr 18 heterozygosity, resulting in the production of adenoma cells homozygous for the mutant *Apc* allele [24, 25]. In trans-*Apc*^+/−^*Cdx1*^+/−^ mice, adenoma cells possessed only the mutant *Apc* allele and the wild-type (wt) *Cdx1* allele, which rendered genetic analysis of the role of Cdx1 in *Apc*^+/−^ mice challenging [15, 26]. Thus, a heterozygous *Cdx1* mutation did not produce a phenotype in trans-*Apc*^+/−^*Cdx1*^+/−^ mice, and only weak invasiveness was observed in *Apc*^+/−^*-Cdx2*^f/f^*-*Villin-CreER^T^ mouse [15].

CDX1/2 can inhibit colon cancer cell proliferation [10, 27, 28], and play roles in the stem cell function of normal intestinal epithelial cells and in colon cancer cell differentiation [29, 30]. CDX1/2 also decrease the reporter activity in TOPflash assays [28, 31]. These results suggest that CDX1/2 suppress β-catenin–T-cell factor (TCF) transcriptional activity and colon cancer stemness. However, there is no direct evidence of the regulation of colon cancer stemness and of expression of β-catenin-target genes by CDX1/2 (along with the underlying mechanisms). CDX2 binds to genomic regions bound by TCF4 as well and is necessary for the expression of TCF-target genes [32, 33]. However, CDX1/2 and β-catenin–TCF4 complex exhibit antagonistic functions, potentially inhibiting each other within the shared genome of colon cancer cells. In this study, we analyzed the roles of CDX1/2 in the malignant progression of colon cancer, regulation of cancer stemness, and β-catenin-mediated transcription in colon cancer cells.

## Results

### Malignant progression in intestinal tumors induced by *Cdx1* and *Cdx2* deletion mutations

**Fig. 1:**
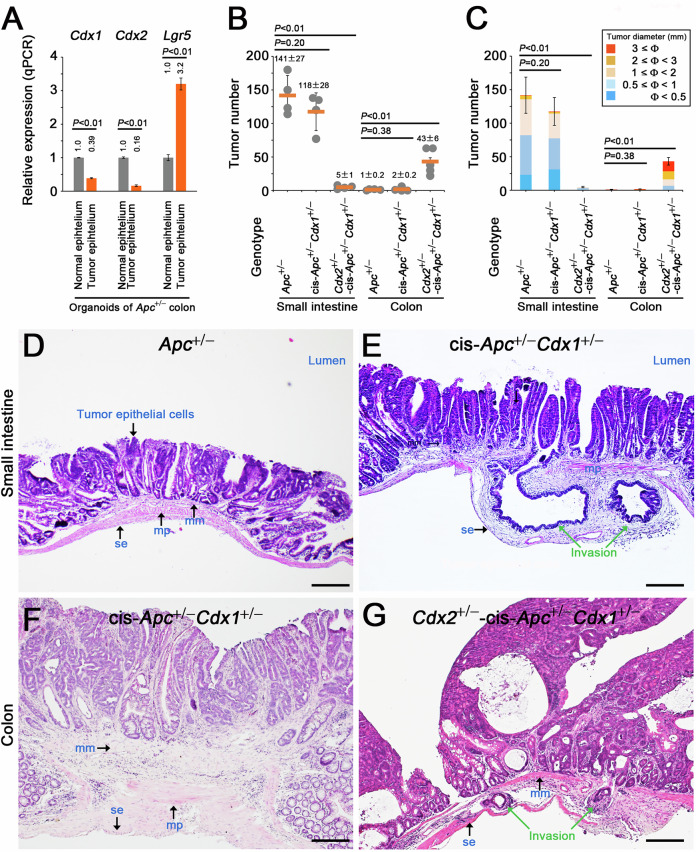
Suppression of malignant progression in colonic tumorigenesis by Cdx1 and Cdx2. **A** qPCR data showing the relative expression (mean ± SD) of *Cdx1*, *Cdx2*, and *Lgr5* in colonic tumor organoids derived from *Apc*^+/−^ mice (compared with those in normal epithelium). *P*-values were calculated using a Student′s *t*-test (**A**–**C**). Number (**B**) and size (**C**) of intestinal tumors in *Apc*^+/−^, cis-*Apc*^+/−^*Cdx1*^+/−^, and *Cdx2*^+/−^-cis-*Apc*^+/−^*Cdx1*^+/−^ mice at 10–12 weeks of age (mean ± SD; n = 4–5, excluding dysplastic crypts). Hematoxylin and eosin (H&E)-stained small intestinal tumors in *Apc*^+/−^ (**D**) and cis-*Apc*^+/−^*Cdx1*^+/−^ (**E**) mice. Green arrows indicate invasive tumor cells (**E**, **G**). Abbreviations in **D**–**G**: mm muscularis mucosa, mp muscularis propria, se serosa. Scale bars, 200 µm (**D**–**G**). H&E-stained colonic tumors from cis-*Apc*^+/−^*Cdx1*^+/−^ (**F**) and *Cdx2*^+/−^-cis-*Apc*^+/−^*Cdx1*^+/−^ (**G**) mice.

*Cdx1* and *Cdx2* mRNA were detectable in the colonic tumor organoids derived from *Apc*^+/−^ mice [25] but were expressed at significantly lower levels than those observed in normal colonic epithelium (Fig. 1A). To delete *Cdx1* in adenomas in *Apc*^+/−^ mice, we subsequently generated cis-*Apc*^+/−^*Cdx1*^+/−^ mice that carried both *Apc* and *Cdx1* deletion mutations [34] on the same Chr 18 homolog via meiotic recombination. The loss of wt-*Cdx1* and wt-*Apc* in colonic tumor organoids derived from cis-*Apc*^+/−^*Cdx1*^+/−^ mice was confirmed by PCR (Fig. S1A). We also crossed cis-*Apc*^+/−^*Cdx1*^+/−^ mice with *Cdx2*^+/−^ mice. The loss of *Cdx1* did not affect the number or size of intestinal tumors in *Apc*^+/−^ mice (Fig. 1B, C and Fig. S1B–D). In contrast, the *Cdx2* mutation resulted in increased number of colonic tumors and formation of large tumors that were not observed in *Apc*^+/−^ and cis-*Apc*^+/−^*Cdx1*^+/−^ mice (Fig. 1B, C and Fig. S1D), as reported previously [10]. Tumor epithelial cells in the small intestine of cis-*Apc*^+/−^*Cdx1*^+/−^ mice deeply invaded the submucosa (Fig. 1D, E and Fig. S2A–C, and Table S1). Although no invasion was observed in case of *Apc*^+/−^*Cdx1*^+/−^ colonic tumors (Fig. 1F), an additional *Cdx2* mutation induced submucosal invasion (Fig. 1G and Fig. S2D). These results provide genetic evidence that Cdx1, cooperatively with Cdx2, suppressed the malignant progression of intestinal tumors.

### Suppression of colon cancer stemness by CDX1 and CDX2

**Fig. 2:**
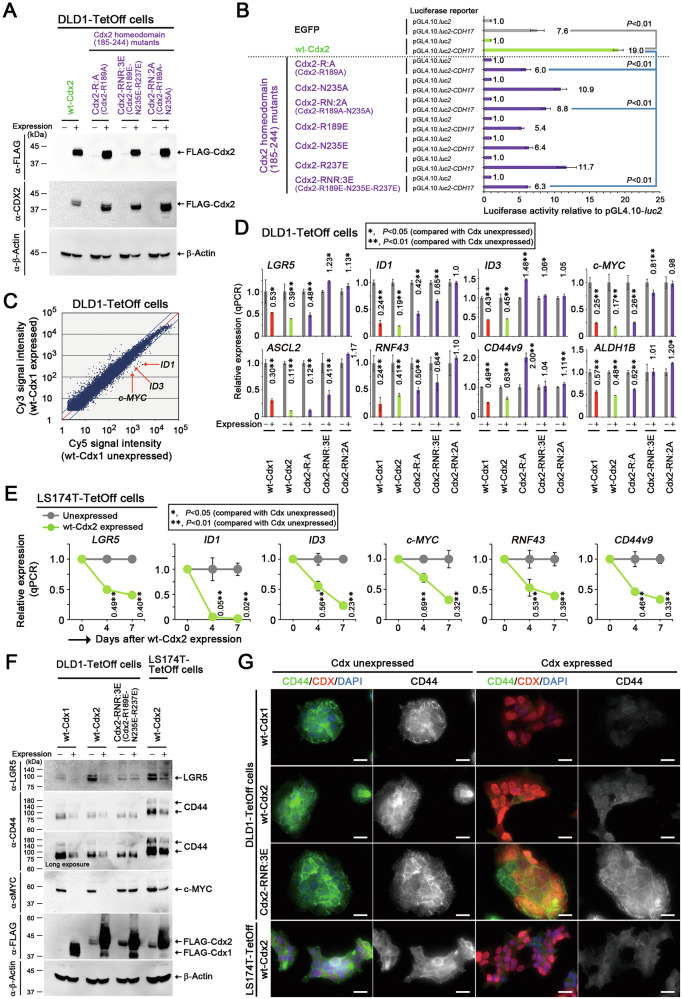
Suppression of colon cancer cell stemness by CDX1 and CDX2. **A** Immunoblots showing doxycycline-controlled inducible expression of FLAG-wt-Cdx2 or its homeodomain (HD) mutants in DLD1-TetOff cells. β-Actin was a loading control (**A**, **F**). **B** *CDH17 luc* reporter activities (mean ± SD) relative to those of the pGL4.10-*luc2* control upon expressing wt-Cdx2 or its HD mutants. *P*-values were calculated using a Student′s *t*-test (**B**, **D**, **E**). **C** Microarray data showing the gene expression profiles of DLD1-TetOff cells after expression of wt-Cdx1 (Cy3) for 12 h (compared with those of cells not expressing wt-Cdx1; Cy5). Red arrows denote the relationship between cells with Cdx1 expression and without Cdx1 expression in terms of *ID1*, *ID3*, and *c-MYC* expression. qPCR data showing the relative expression (mean ± SD) of colon cancer stemness-related genes upon expressing wt-Cdx1, wt-Cdx2, or Cdx2 HD mutants for 2 days in DLD1-TetOff cells (**D**) and for 4 and 7 days in LS174T-TetOff cells (**E**), when compared with cells without their expression. **F** Immunoblots showing the expression of LGR5, CD44, and c-MYC upon expressing wt-Cdx1, wt-Cdx2, or Cdx2-RNR:3E in DLD1-TetOff and LS174T-TetOff cells. Note that the basal expression levels of LGR5 were different among the TetOff clones. **G** Immunocytochemistry showing the expression of CD44 (green) and Cdx1/2 (red) upon expression of wt-Cdx1, wt-Cdx2, or Cdx2-RNR:3E in DLD1-TetOff and LS174T-TetOff cells. The nuclei were stained with DAPI (blue). Scale bars, 20 µm.

We analyzed the gene expression profiles after expressing mouse wt-Cdx1 or wt-Cdx2 in human colon cancer-derived DLD1-TetOff cells (Fig. 2A, B and Fig. S3A). Microarray analysis showed that 12 and 24 h after expressing wt-Cdx1 and wt-Cdx2, two members of the inhibitor of DNA binding (ID) family (*ID1* and *ID3*), as well as *c-MYC* were downregulated by 60-90% in DLD1-TetOff cells (Fig. 2C and Table S2). Similar results were observed via qPCR (Fig. 2D, DLD1-TetOff cells; Fig. 2E, LS174T-TetOff cells). Reduced c-MYC expression at the protein level was also confirmed following expression of wt-Cdx1 and wt-Cdx2 (Fig. 2F). Notably, previous studies have shown that ID1, ID3, and c-MYC help maintain the self-renewal capacity of colon cancer stem cells [22, 35, 36]. Concordantly, the expression of cancer stemness-related genes, such as *LGR5* and *CD44*, was significantly reduced at the mRNA and protein levels in TetOff cells upon expressing wt-Cdx1/2 (Fig. 2D–F and Fig. S3B, C). This expression was also reduced upon expression of a transcription-deficient homeodomain (HD) mutant of Cdx2, i.e., Cdx2-R189A (Cdx2-R:A) but not by the other HD mutants, i.e., Cdx2-R189E-N235E-R237E (Cdx2-RNR:3E) and Cdx2-R189A-N235A (Cdx2-RN:2A) (Fig. 2B, D, F and Fig. S3B). Cell-surface expression of CD44 also decreased in TetOff cells upon expressing wt-Cdx1/2 (Fig. 2G and Fig. S4A, B, D), but not by Cdx2-RNR:3E (Fig. 2G and Fig. S4C). Amino acid residues 189, 235, and 237 in Cdx2 HD and their corresponding residues 158, 204, and 206 in Cdx1 HD were found to be critical for DNA binding [27, 37]. These results suggest that CDX1/2 suppressed the expression of genes essential for colon cancer stemness through their HDs.

### Increased colon cancer stemness by *Cdx1* and *Cdx2* deletion mutations

**Fig. 3:**
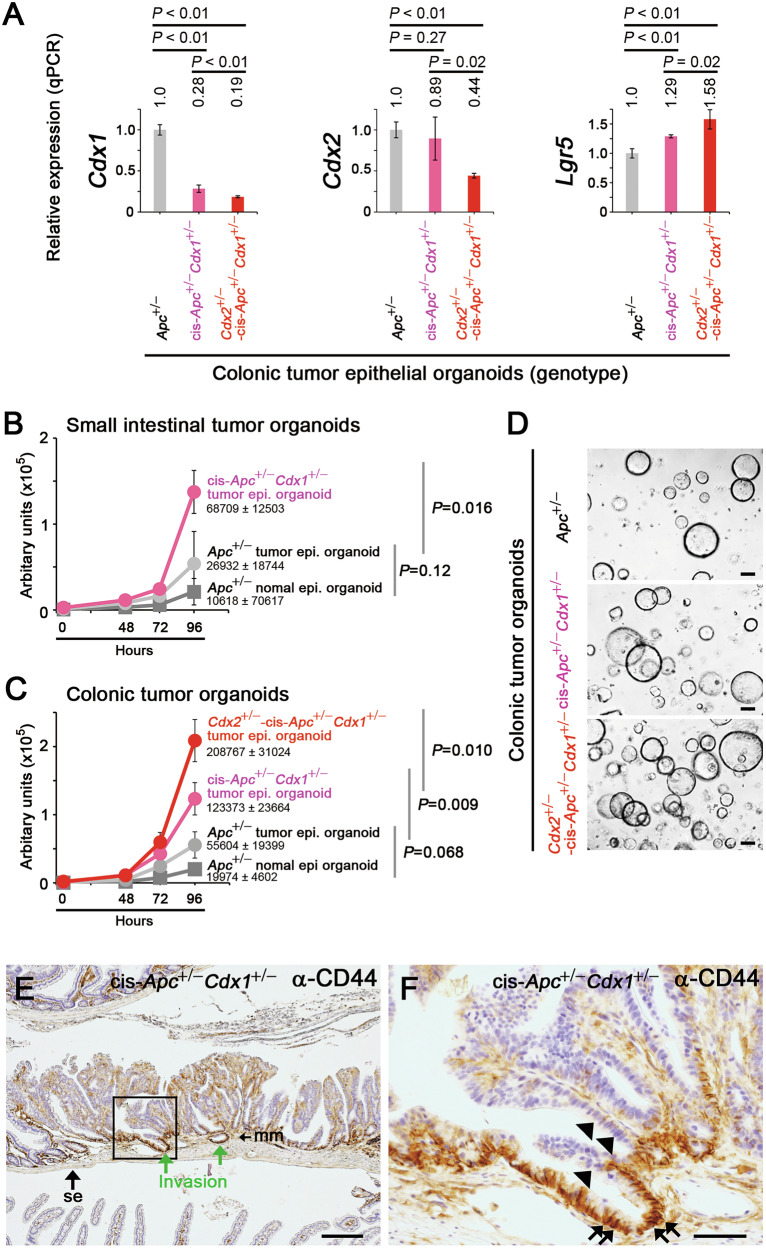
Increased intestinal tumor stemness by *Cdx1* and *Cdx2* mutations. **A** qPCR data showing the relative expression (mean ± SD) of *Cdx1, Cdx2*, and *Lgr5* in colonic tumor organoids derived from cis-*Apc*^+/−^*Cdx1*^+/−^ and *Cdx2*^+/−^-cis-*Apc*^+/−^*Cdx1*^+/−^ mice (compared with those from *Apc*^+/−^ mice). *P*-values were calculated using a Student′s *t*-test (**A**–**C**). Growth rate (mean ± SD) of organoid cells in normal and tumor epithelial tissues derived from the small intestine (**B**) and colon (**C**) of *Apc*^+/−^, cis-*Apc*^+/−^*Cdx1*^+/−^, and *Cdx2*^+/−^-cis-*Apc*^+/−^*Cdx1*^+/−^ mice. **D** Tumor organoids derived from *Apc*^+/−^, cis-*Apc*^+/−^*Cdx1*^+/−^, and *Cdx2*^+/−^-cis-*Apc*^+/−^*Cdx1*^+/−^ colonic tumors. Scale bars, 100 µm. **E**, **F** Immunohistochemistry of Cd44 expression (brown) in a small intestinal tumor of a cis-*Apc*^+/−^*Cdx1*^+/−^ mouse. **F** shows the magnified image of the boxed region in (**E**). The arrows in **F** indicate cells with elevated Cd44 expression, while arrowheads indicate cells with lower Cd44 expression. The tissue was also counterstained with hematoxylin (blue). Scale bars, 100 µm (**E**) and 20 µm (**F**).

In colonic tumor organoids derived from cis-*Apc*^+/−^*Cdx1*^+/−^ and *Cdx2*^+/−^*-*cis-*Apc*^+/−^*Cdx1*^+/−^ mice, the expression of *Cdx1* and *Cdx2* was lower than that in *Apc*^+/−^ tumor organoids, whereas that of *Lgr5* was higher (Fig. 3A). Similarly, higher proliferation rates were observed in the small-intestinal tumor organoids derived from cis-*Apc*^+/−^*Cdx1*^+/−^ mice (Fig. 3B) and in colonic tumor organoids derived from cis-*Apc*^+/−^*Cdx1*^+/−^ and *Cdx2*^+/−^*-*cis-*Apc*^+/−^*Cdx1*^+/−^ mice than in those derived from *Apc*^+/−^ mice (Fig. 3C, D). These results indicate that reduced expression of Cdx1/2 promoted colon cancer stemness.

The β-catenin-target gene, *Cd44*, is a marker of cancer stemness [38] and has been implicated in tumor invasion [39]. Cd44 was upregulated in the leading cells at the invasive front of the intestinal tumors in cis-*Apc*^+/−^*Cdx1*^+/−^ mice (Fig. 3E, F and Fig. S5A–K). In contrast, Cd44 expression was lower in cells on the opposite side (Fig. 3E, F and Fig. S5A–K) and in cells in the deeply invaded region (Fig. S5L). These results suggest that a cancer-stem cell feature mediated invasion during colonic tumorigenesis. Proliferating cells were also observed in the non-invasive and invasive regions of cis-*Apc*^+/−^*Cdx1*^+/−^ intestinal tumors (Fig. S5M–O). The role of increased proliferation through CDX1/2 suppression during invasion remains to be further investigated.

### Reduction of RNA polymerase II (Pol II), DRB sensitivity-inducing factor (DSIF), and RNA polymerase II-associated factor 1 (PAF1) levels around the TSS of *LGR5* induced by CDX1 and CDX2

**Fig. 4:**
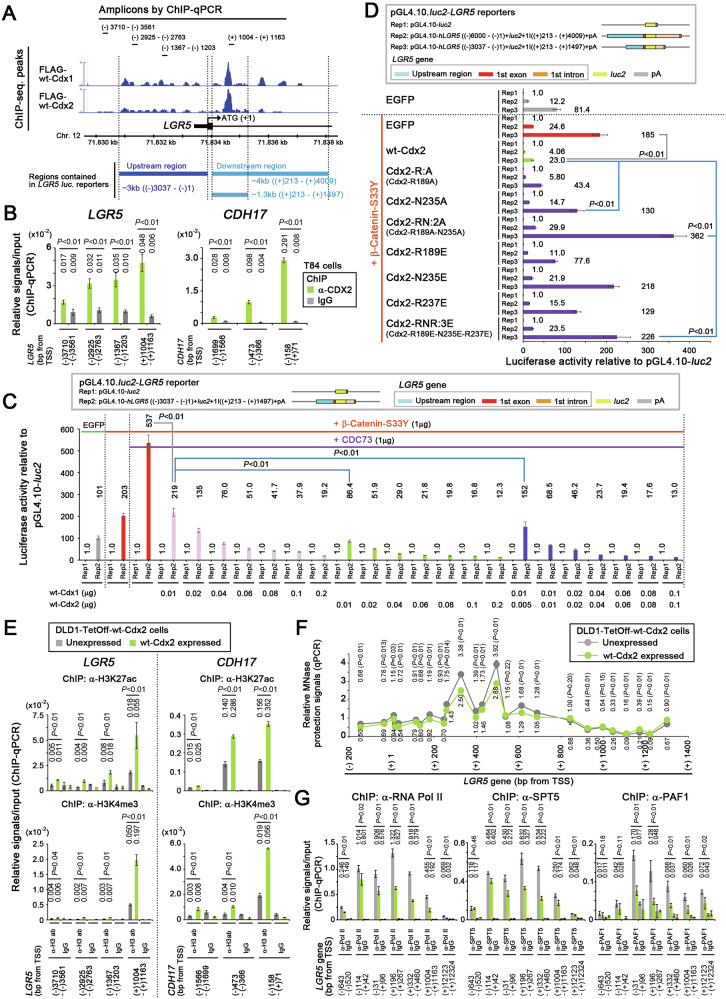
Decreased occupancy levels of Pol II, SPT5, and PAF1 on *LGR5* upon CDX2 expression. **A** Integrative genome-viewer window showing the occupancy of FLAG-tagged wt-Cdx1 and wt-Cdx2 on the *LGR5* gene in DLD1-TetOff cells. Genomic regions analyzed by ChIP-qPCR in **B** are indicated above, whereas those cloned in the *LGR5 luc* reporter **C**, **D** are indicated below. **B** ChIP-qPCR data showing the relative occupancy (mean ± SD) of endogenous CDX2 at the indicated positions of *LGR5* and CDX2-target gene *CDH17* (used as a positive control) in T84 cells. *P*-values were calculated using a Student′s *t*-test (**B**–**G**). **C** *LGR5 luc* reporter activities (mean ± SD) relative to those of the pGL4.10-*luc2* control upon expressing wt-Cdx1, wt-Cdx2, or both wt-Cdx1 and wt-Cdx2. The amounts of transfected plasmid DNA used to overexpress wt-Cdx1 and wt-Cdx2 are indicated below the bar graph. **D** *LGR5 luc* reporter activities (mean ± SD) relative to those of the pGL4.10-*luc2* control upon expressing wt-Cdx2, or its homeodomain (HD) mutants, or β-catenin-S33Y. **E** ChIP-qPCR data showing the relative occupancy (mean ± SD) of H3K27ac and H3K4me3 at the indicated positions of *LGR5* and *CDH17* after expressing wt-Cdx2 in DLD1-TetOff cells for 1 day. **F** qPCR data quantifying MNase protection assays reflecting the chromatin architecture at the indicated positions in *LGR5* after expressing wt-Cdx2 in DLD1-TetOff cells for 1 day. **G** ChIP-qPCR data showing the relative occupancy (mean ± SD) of Pol II, SPT5, and PAF1 at the indicated positions in *LGR5* after expressing wt-Cdx2 in DLD1-TetOff cells for 1 day.

To elucidate the mechanism whereby CDX1/2 suppressed *LGR5* expression, we conducted a chromatin immunoprecipitation-sequencing (ChIP-seq) analysis of FLAG-tagged wt-Cdx1/2 variants expressed in DLD1-TetOff cells. A significant peak was observed approximately 1000 base pairs (bp) downstream of the *LGR5* transcription start site (TSS; Fig. 4A), which was confirmed by ChIP-qPCR for both endogenous CDX2 (Fig. 4B) and exogenously expressed wt-Cdx1/2 (Fig. S6A, B). In contrast, the binding of wt-Cdx2 to the *LGR5* gene was weakened by the R189A-N235A mutation (Fig. S6C, Cdx2-RN:2A). We recently constructed *LGR5* luciferase (*luc*) reporter plasmids [40], wherein *luc* was flanked by Cdx1/2 binding regions upstream and downstream of the *LGR5* TSS (Fig. 4A, C). As reported [40], a stable β-catenin mutant with an S33Y substitution increased the *LGR5 luc* reporter activity (Fig. 4C). In contrast, *LGR5 luc* reporter activity was suppressed by wt-Cdx1/2 in a dose-dependent manner; the activity decreased from 537 to 219 (41%) and from 537 to 86.4 (16%) after transfecting H293T cells with 0.01 μg of a Cdx1-expression vector (plasmid-Cdx1) or a plasmid-Cdx2, respectively (Fig. 4C). *LGR5 luc* reporter activity decreased from 537 to 68.5 (13%) after co-transfecting 0.01 μg each of plasmid-Cdx1 and plasmid-Cdx2 (Fig. 4C). These results suggest that CDX1 and CDX2 cooperated to suppress *LGR5* expression in an additive manner (rather than synergistically). *LGR5 luc* reporter activity was also suppressed by Cdx2-R:A but not by Cdx2-RN:2A, Cdx2-RNR:3E, or other Cdx2 HD mutants (Fig. 4D). Similar results were obtained for the Cdx1 HD mutants (Fig. S6D). As no significant differences were found between the functions of CDX1 and CDX2, we mainly analyzed CDX2 in subsequent experiments.

Expressing wt-Cdx2 increased H3K27ac and H3K4me levels around the promoter region and first intron of both *LGR5* and CDX2-target gene *CDH17* (Fig. 4E) [41]. Even after wt-Cdx2 expression, closed chromatin structures were not observed around the *LGR5* TSS, as determined in the micrococcal nuclease-protection assays (Fig. 4F). We then analyzed the dynamics of Pol II and its-associated factors, including SPT5, a component of DSIF complex [42], and PAF1, a component of RNA polymerase-associated factor 1 complex (PAF1C) [43]. These molecules are essential for promoter-proximal pausing and regulate mRNA transcription [43–46]. Wt-Cdx1/2 decreased the occupancy levels of Pol II, SPT5, and PAF1 downstream of the *LGR5* TSS (Fig. 4G and Fig. S6E); however, a similar result was not obtained with Cdx2-RN:2A (Fig. S6F). These results suggest that CDX1/2 suppressed Pol II-mediated elongation from the promoter-proximal region of *LGR5* through HD.

### Suppression of β-catenin-facilitated active Pol II complex formation by CDX1/2 through DSIF and PAF1C

**Fig. 5:**
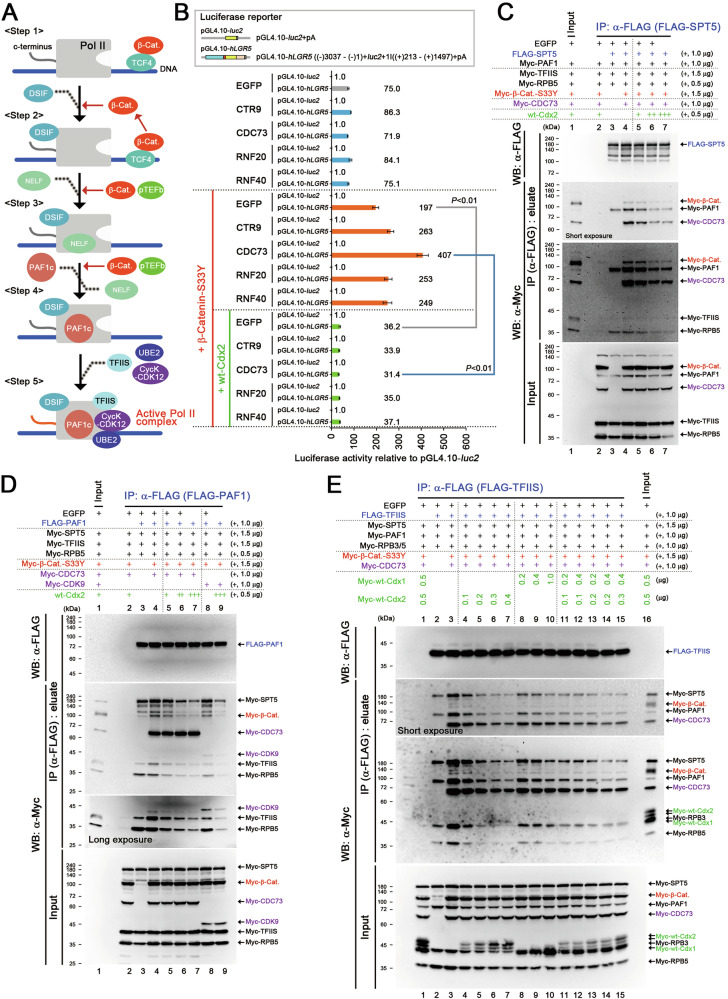
Suppression of Pol II–DSIF–PAF1C complex formation by CDX1/2. **A** Mechanism of β-catenin-facilitated formation of the active Pol II complex through PAF1C. *Step 1*: TCF4 recruits β-catenin to its target genes; *Step 2*: β-catenin recruits the DSIF complex to Pol II; *Step 3*: β-catenin facilitates the formation of the Pol II–DSIF–NELF complex; *Step 4*: β-catenin facilitates the formation of the Pol II–DSIF–PAF1C complex; *Step 5*: The Pol II–DSIF–PAF1C complex forms a complex with TFIIS, UBE2, and cyclin K (CycK)-CDK12 and initiates mRNA transcription. **B** *LGR5 luc* reporter activities (mean ± SD) relative to those of the control pGL4.10-*luc*2 upon expressing PAF1C components, β-catenin-S33Y, and wt-Cdx2. *P*-values were calculated using a Student′s *t*-test. Immunoprecipitation (IP) assays showing the effect of wt-Cdx2 on β-catenin-S33Y (β-Cat.)-facilitated complex formation of **C** SPT5 (containing PAF1, TFIIS, and RPB5) and **D** PAF1 (containing SPT5, TFIIS, and RPB5). In the IP assays, the indicated proteins were co-expressed with either FLAG-SPT5 (**C**) or FLAG-PAF1 (**D**). The co-immunoprecipitated Myc-tagged proteins were analyzed using immunoblotting. The amounts of plasmid DNA transfected are indicated on the right. **E** IP assays showing the effects of wt-Cdx1, wt-Cdx2, or both wt-Cdx1 and Cdx2 on β-catenin-S33Y (β-Cat.)-facilitated formation of a complex involving TFIIS, SPT5, PAF1, and RPBs. The indicated proteins were expressed in the IP assays along with FLAG-TFIIS. The co-immunoprecipitated Myc-tagged proteins were analyzed via immunoblotting. The amounts of transfected plasmid DNA used for expressing wt-Cdx1 and wt-Cdx2 are indicated above the gel images.

We recently showed that β-catenin regulated *LGR5* expression through DSIF and PAF1 complexes (Fig. 5A) [40]. As reported in this study [40], β-catenin-S33Y increased *LGR5 luc* reporter activity cooperatively with PAF1C components (Fig. 5B). In contrast, wt-Cdx1/2 suppressed *LGR5 luc* reporter activity (Fig. 5B). Interestingly, wt-Cdx1/2 interacted with PAC1C components (Fig. S7A, B). We then analyzed the role of CDX1/2 in regulating the formation of active Pol II complex via PAF1C by expressing essential components, including SPT5 from DSIF; PAF1 and CDC73 from PAF1C; and RPB2, 3, and 5 from PoI II; and TFIIS (Fig. 5A), based on previously reported findings [40]. In this experiment, we hypothesized that the amount of TFIIS bound to SPT5 or PAF1C correlated with the formation of active Pol II complex (Fig. 5A) [40].

We expressed FLAG-SPT5 along with Myc-tagged variants of PAF1, TFIIS, and RPBs. β-Catenin-S33Y increased the binding of FLAG-SPT5 to Myc-tagged RPB2, PAF1, and TFIIS with the cooperation with CDC73 (Fig. 5C, lane 4), whereas wt-Cdx2 impaired those interactions (Fig. 5C, lanes 5–7). Similarly, β-catenin-S33Y increased the binding of FLAG-PAF1 to Myc-tagged RPB2, SPT5, and TFIIS with the cooperation with CDC73 or CDK9 (Fig. 5D, lanes 4, 8), whereas wt-Cdx2 suppressed such binding (Fig. 5D, lanes 5-7, 9). Additionally, wt-Cdx1/2 suppressed the β-catenin-S33Y-induced increase in the binding of FLAG-TFIIS to Myc-tagged SPT5, PAF1, and RPBs in a dose-dependent manner (Fig. 5E, lanes 4–7, 8–10). Transfection of 0.1 μg plasmid-Cdx2 suppressed the formation of the Pol II–DSIF–PAF1C complex to a similar extent as transfection of 0.2 μg plasmid-Cdx1 (Fig. 5E, lanes 4, 8). It is noted that both plasmid-Cdx2 and plasmid-Cdx1 increased the *CDH17 luc* reporter activity to a similar extent (Fig. 2B and Fig. S3A). These results suggest that Cdx2 had an approximately 2-fold more potent inhibitory effect on complex formation than Cdx1, consistent with the results above (Fig. 4C). Co-transfection of 0.1 μg plasmid-Cdx2 and 0.2 μg plasmid-Cdx1 resulted in a similar inhibitory effect on complex formation as transfection of 0.2 μg plasmid-Cdx2 alone (Fig. 5E, lanes 5, 11). Collectively, these results indicate that CDX1/2 additively suppressed β-catenin-facilitated formation of the active Pol II complex by inhibiting the binding of DSIF and PAF1C to Pol II and that the combined levels of CDX1/2 determined the extent of suppression.

### Suppression of β-catenin-facilitated Pol II–DSIF–PAF1C complex formation by CDX2 was abrogated by a mutation in its HD

**Fig. 6:**
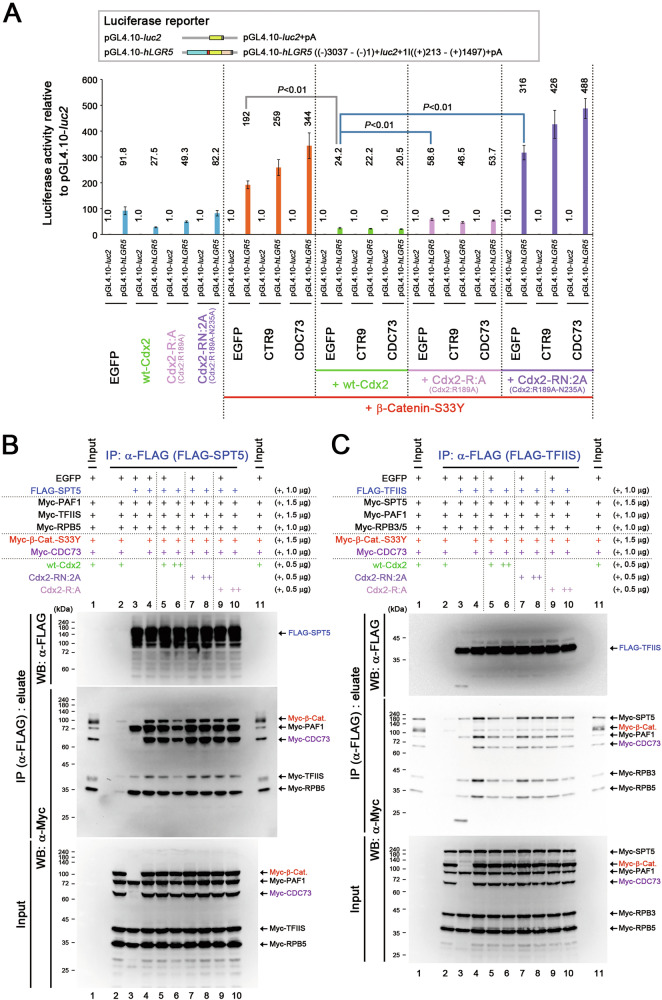
CDX2-mediated suppression of Pol II–DSIF–PAF1C complex formation blocked by mutations of its homeodomain. **A** *LGR5 luc* reporter activities (mean ± SD) relative to those of the pGL4.10-*luc*2 control upon expressing β-catenin-S33Y, PAF1C components, wt-Cdx2, and its homeodomain (HD) mutants. *P*-values were calculated using a Student′s *t*-test. IP assays showing the effects of wt-Cdx2 and its HD mutants on β-catenin-S33Y (β-Cat.)-facilitated complex formation: **B** SPT5-containing complexes with PAF1, TFIIS, and RPB5 or **C** TFIIS-containing complexes with SPT5, PAF1, and RPBs. In the IP assays, the indicated proteins were expressed along with FLAG-SPT5 (**B**) or FLAG-TFIIS (**C**).

Consistent with the observation that the Cdx2-RN:2A or Cdx2-RNR:3E mutant did not reduce *LGR5* expression at the mRNA level (Fig. 2D, F), *LGR5 luc* reporter activity was not suppressed by Cdx2-RN:2A, Cdx1-RN:2A or Cdx1-RNR:3E (Fig. 6A and Fig. S6D). However, *LGR5 luc* reporter activity was suppressed by Cdx2-R:A and Cdx1-R:A, albeit less effectively than that by wt-Cdx1/2 (Fig. 6A and Fig. S6D). Likewise, Cdx2-RN:2A or Cdx1-RNR:3A did not reduce the binding of FLAG-SPT5 to Myc-tagged RPB2 or PAF1, or the binding of FLAG-TFIIS to Myc-tagged SPT5, PAF1, and RPBs in the presence of β-catenin-S33Y and CDC73 (Fig. 6B, C and Fig. S8A, lanes 7, 8). However, the Cdx2-R:A and Cdx1-R:A mutants did suppress such binding, although less effectively than that by wt-Cdx2 and wt-Cdx1 (Fig. 6B, C and Fig. S8A, lanes 5, 6, 9, 10). These results suggest that the HD of CDX1/2 helped suppressed β-catenin-facilitated formation of the active Pol II complex through a mechanism independent of transcriptional activation.

### Suppression of β-catenin-facilitated Pol II–DSIF–PAF1C complex formation by CDX2 mediated by its N-terminus and HD

**Fig. 7:**
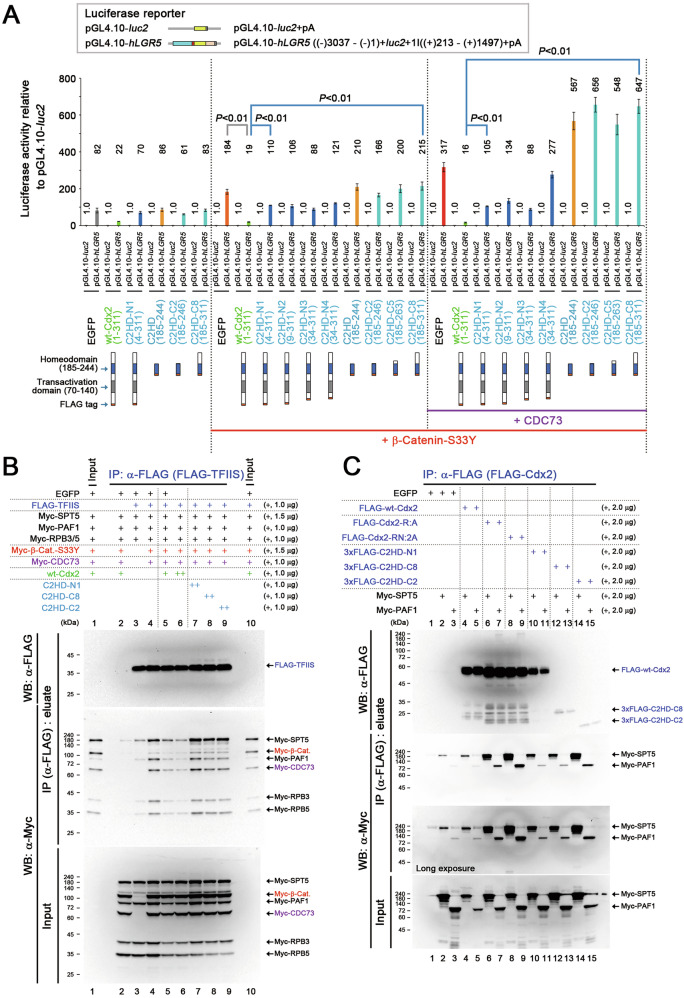
Contribution of the CDX2 homeodomain to the suppression of Pol II–DSIF–PAF1C complex formation. **A** *LGR5 luc* reporter activities (mean ± SD) relative to those of the pGL4.10-*luc*2 control upon expressing β-catenin-S33Y, CDC73, and wt-Cdx2 and its deletion mutants. *P*-values were calculated using a Student′s *t*-test. **B** IP assays showing the effects of wt-Cdx2 and its deletion mutants on the β-catenin-S33Y (β-Cat.)-facilitated formation of a complex involving TFIIS, SPT5, PAF1, and RPBs. **C** IP assays showing the interaction between SPT5 and Cdx2 (or its mutants) and between PAF1 and Cdx2 (or its mutants).

To delineate the regions of CDX2 responsible for suppressing β-catenin-facilitated Pol II activation, we further employed Cdx2-deletion mutants harboring its HD (Fig. 7A). Mutant C2HD contained HD, and mutant C2HD-C2 contained HD and its C-terminally adjacent lysyl-lysine (KK) domain that aids nuclear localization [27]. C2HD-N mutants contained HD with C-terminal KK, and different N-terminal extensions harboring the transactivation domain [27, 47]. C2HD-C mutants contained HD and different C-terminal extensions. Deletion of the N-terminal region impaired the Cdx2-induced suppression of *LGR5* reporter activity (Fig. 7A, C2HD-N1). Consistently, the N-terminal deletion mutants of Cdx2 did not reduce the β-catenin-S33Y-induced FLAG-TFIIS-bound Myc-tagged SPT5, PAF1, and RPBs levels (Fig. 7B, lane 7). Interestingly, HD mutations enhanced the interactions of Cdx1/2 with PAF1 and SPT5 complexes (Fig. 7C, lanes 6–9 and Fig. S9A, lanes 9–14). Likewise, the HD interacted with PAF1 and SPT5 complexes (Fig. 7C, lanes 14, 15). Therefore, the N-terminal domain and HD of CDX1/2 mainly contributed to the suppression of β-catenin-facilitated formation of the active Pol II complex.

### Suppression of the interactions of β-catenin with DSIF and PAF1C by CDX1 and CDX2

**Fig. 8:**
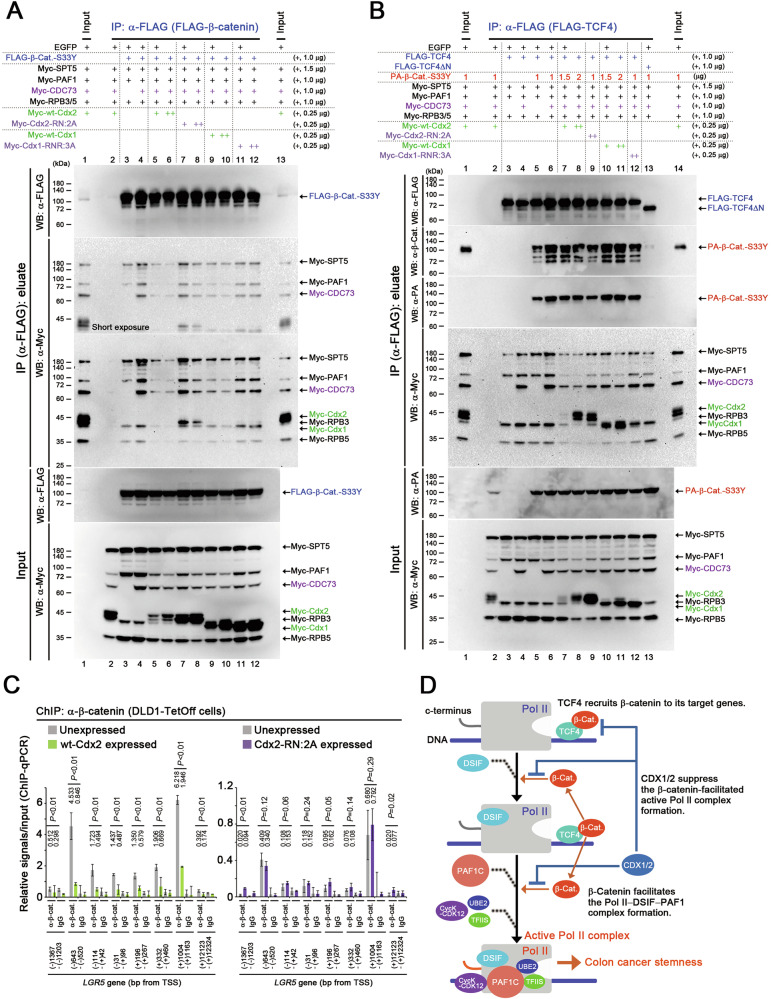
Suppression of the interaction between β-catenin and PAF1C by CDX1 and CDX2. **A** IP assays showing the effects of wt-Cdx1/2 and their homeodomain (HD) mutants on the interaction of β-catenin-S33Y (β-Cat.) with SPT5, PAF1 components, and RPBs. The indicated proteins were expressed in the IP assays along with FLAG-β-catenin-S33Y. **B** IP assays showing the effects of wt-Cdx1/2 and their HD mutants on the interaction of FLAG-TCF4 with PA-β-catenin-S33Y (β-Cat.), Myc-tagged SPT5, PAF1 components, and RPBs. In the IP assays, the indicated proteins were expressed along with FLAG-TCF4. **C** ChIP-qPCR data showing the relative occupancy (mean ± SD) of β-catenin at the indicated positions in *LGR5* after expressing wt-Cdx2 or Cdx2-RN:2A for 1 day in DLD1-TetOff cells, when compared with that in cells with Cdx unexpressed. *P*-values were calculated using a Student′s *t*-test. **D** Transcriptional mechanism underlying the inhibition of stable β-catenin by CDX1/2 via DSIF and PAF1C complexes, resulting in the suppression of colon cancer stemness. First, TCF4 recruits β-catenin to its target gene. β-Catenin then recruits DSIF and PAF1 complexes to the Pol II complex to facilitate the formation of the active Pol II complex, which promotes colon cancer stemness. CDX1/2 suppress these processes. DSIF and PAF1 complexes act as platforms that integrate and funnel oncogenic and tumor-suppressive signals into gene expression, thereby controlling cancer stemness.

We further examined the effects of CDX1/2 on the interactions between β-catenin and Pol II, DSIF, and PAF1 complexes. To this end, FLAG-tagged β-catenin-S33Y was co-expressed with Myc-tagged SPT5, PAF1, CDC73, and RPBs (Fig. 8A). Wt-Cdx1/2 suppressed the interaction between β-catenin and the SPT5, PAF1, and Pol II complexes (Fig. 8A, lanes 5, 6, 9, 10), whereas Cdx2-RN:2A and Cdx1-RNR:3A mutations weakened this suppression by wt-Cdx1/2 (Fig. 8A, lanes 7, 8, 11, 12 and Fig. S10A–C).

We further expressed FLAG-tagged TCF4 along with PA-tagged β-catenin-S33Y, Myc-tagged SPT5, and PAF1C components. β-Catenin-S33Y and CDC73 increased the SPT5 and PAF1C levels in the TCF4 complex (Fig. 8B, lanes 5, 6 and Fig. S10D) but not in a TCF4ΔN mutant lacking the domain required for β-catenin binding (Fig. 8B, lane 13). Due to reduced levels of PA-tagged β-catenin-S33Y in the presence of Cdx1/2 (Fig. S10D), we increased the amount of the transfected plasmid DNA expressing PA-tagged β-catenin-S33Y in cells expressing wt-Cdx1/2 (Fig. 8B, lanes 7, 8, 10, 11). Wt-Cdx1/2 significantly reduced the β-catenin-S33Y- and CDC73-induced SPT5 and PAF1C levels in the TCF4 complex (Fig. 8B, lanes 7, 8, 10, 11), whereas Cdx2-RN:2A and Cdx1-RNR:3A mutations weakened the effects of wt-Cdx1/2 (Fig. 8B and Fig. S10D, lanes 9, 12). Wt-Cdx1/2 also reduced β-catenin levels in the TCF4 complex (Fig. 8B and Fig. S10D), consistent with an earlier study [28]. Likewise, wt-Cdx1/2 reduced the amount of β-catenin bound to *LGR5*, whereas Cdx2-RN:2A did not (Fig. 8C and Fig. S10E). These results suggest that CDX1/2 suppressed β-catenin-facilitated formation of the active Pol II complex by disrupting the interactions between β-catenin and the DSIF and PAF1 complexes and those between β-catenin and TCF4 (Fig. 8D).

## Discussion

The complete loss of *Cdx1* resulted in extensive invasion of the small-intestinal tumor cells, whereas the absence of *Cdx1* in conjugation with a heterozygous *Cdx2* mutation led to the invasion of the colonic tumor cells (Fig. 1). Organoids derived from colonic tumors carrying *Cdx1* and *Cdx2* mutations exhibited increased proliferation and elevated *Lgr5* expression levels (Fig. 3). Additionally, we analyzed data hosted on the Gene Expression Omnibus (GEO) Database and compared the gene expression levels between *LGR5*^high^ and *LGR5*^low^ cells from five colorectal cancer (CRC) human organoid clones [48]. In four out of five human CRC organoid clones, the levels of either both *CDX1* and *CDX2*, or one of the genes, were lower in *LGR5*^high^ cells than in those in *LGR5*^low^ cells, with no overall increases observed in *CDX1/2* expression (Table S3). In the remaining clone, the level of *CDX2* expression decreased to 0.79, while that of *CDX1* increased to 1.18 in *LGR5*^high^ cells (Table S3). Moreover, recent data show that in the stem cells of human colon epithelial cells, the levels of *CDX1* and *CDX2* are reduced to 0.07 and 0.22, respectively (compared with normal epithelial colon cells), while those of *LGR5* increased to 3.48 [49]. Collectively, these results suggest that CDX1/2 helped suppress stemness in both normal and cancerous colon epithelial cells of human and mouse origin. Cdx1/2 cooperatively and additively suppressed β-catenin-induced *LGR5 luc* reporter activity and active Pol II complex formation (Figs. 4 and 5). These findings further suggest that the overall levels of CDX1 and CDX2 determine the suppressive effect on the stemness of colon cancer cells.

Cdx2 bound to the *LGR5* gene and increased the levels of H3K27ac and H3K4me3, similar to its target gene, *CDH17*, resulting in an open chromatin structure around the *LGR5* TSS (Fig. 4). These results are consistent with previous data that showed that Cdx2 contributed to the elevated levels of H3K27ac and the expression of intestinal epithelium-specific genes [50]. Therefore, CDX2 may play a role in enhancing the H3K27ac levels and promoting an open chromatin structure around intestinal epithelium-specific genes. However, Cdx1/2 suppressed the binding of Pol II, SPT5, and PAF1 to the *LGR5* TSS and reduced *LGR5* expression (Fig. 8D). Collectively, these findings suggest that CDX1/2 suppressed *LGR5* expression by regulating core transcriptional machinery, distinct from epigenetic regulatory mechanisms. Cdx2 is also reported to inhibit DNA repair by interacting with Ku proteins [51], suggesting that CDX1/2 also participate directly in DNA metabolism.

Recently, we reported that expression of stable β-catenin induced *LGR5* transactivation by facilitating the formation of active Pol II complex [40]. CDX1/2 counteracted this β-catenin-facilitated formation of the active Pol II complex by inhibiting the interaction of β-catenin with DSIF and PAF1C (Fig. 8D). The functions of Cdx1/2 were suppressed by Cdx1/2 harboring HD mutations, such as Cdx1-RN:2A, Cdx1-RNR:3A, and Cdx2-RN:2A, but not by Cdx1-R:A or Cdx2-R:A (Figs. 2 and 6). These results suggest that the HDs of CDX1/2 have a different function, separate from transactivation. The alternatively spliced form of CDX2, miniCDX2, has also been reported to exhibit a non-transcriptional function that increases p27^Kip1^ expression but does not inhibit the β-catenin–TCF transcriptional activity [52]. Collectively, these results suggest that CDX1/2 exhibits some non-transcriptional functions.

Recently, we reported that PAF1C plays a crucial role in maintaining the stemness of colon cancer cells by regulating the expression of genes associated with cancer stemness [40]. The results of this study also indicate that CDX1/2 inhibit the DSIF and PAF1C functions, while β-catenin acts as an activator. Therefore, DSIF and PAF1C function as a transcriptional platform that controls colon cancer stemness by integrating and funneling both tumor-suppressive and oncogenic signals into gene expression (Fig. 8D).

## Materials and methods

### cis-*Apc*^+/−^*Cdx1*^+/−^ and *Cdx2*^+/−^-cis-*Apc*^+/−^*Cdx1*^+/−^ mutant mice

*Apc*^+/−^, *Cdx1*^+/−^, *Cdx2*^+/−^, and *Apc*^+/−^*Cdx2*^+/−^ mice were generated as described previously [6, 10, 25]. To generate cis-*Apc*^+/−^*Cdx1*^+/−^ mice, a *Cdx1* null mutation was introduced into *Apc*^+/−^ mutant mice. Considering that both *Cdx1* and *Apc* are located on Chr 18, cis-*Apc*^+/−^*Cdx1*^+/−^ mice were generated via meiotic recombination with both mutations on the same chromosomal homolog. To generate *Cdx2*^+/−^-cis-*Apc*^+/−^*Cdx1*^+/−^ mutant mice, cis-*Apc*^+/−^*Cdx1*^+/−^ mice were crossed with *Cdx2*^+/−^ mice. All animal experiments were approved by the Animal Care and Use Committee of FUKUI University (approval number R04023).

### Number of intestinal tumors

The numbers of intestinal tumors in four to five mice of each mutant strain were determined when the mice were 10–12 weeks of age, as described previously [10].

### TetOff cell clones

Cells were cultured in Dulbecco’s Modified Eagle’s medium supplemented with 5% fetal bovine serum. To generate TetOff cell clones, plasmids were transfected into TetOff cells using Lipofectamine LTX^TM^ Reagent with Plus^TM^ Reagent (15338100; Thermo Fisher Scientific). TetOff cell clones were generated by transfecting the human colon cancer cell lines, DLD1 (CCL-221; ATCC) and LS174T (CL188; ATCC) with pTet-Off- (631017; TAKARA)-based plasmid vectors in accordance with the methodology in a previous study [27, 40]. Gene expression was regulated by adding doxycycline (24390-14-5; TGI), a tetracycline analog, to the culture medium at a final concentration of 0.5 µg/mL. pTRE-Tight (631059; TAKARA)-based plasmid vectors capable of inducible expression of Cdx1, Cdx2, and their HD mutants were transfected into DLD1-TetOff and LS174T-TetOff cells in accordance with a previously published methodology [27, 40]. Both *Cdx1* and *Cdx2* were of mouse origin [27]. These TetOff cells were then exposed to 200 µg/mL hygromycin B gold (ant-hg; InvivoGen) for over 2 weeks after expansion of single-cell clones.

### *Cdx1* and *Cdx2* mutagenesis

*Cdx1* and *Cdx2* were mutated using the PrimeSTAR® Mutagenesis Basal Kit (R046A; TAKARA) and the oligonucleotide primers used for mutagenesis are listed in the Supplementary Materials and Methods. C2HD mutants have been described previously [27].

### DNA microarray analysis

For DNA microarray analysis, RNA was isolated from DLD1-TetOff cells expressing wt-Cdx1 or wt-Cdx2 for 12 and 24 h, using the TRI Reagent® (T9424; Sigma-Aldrich). The cells were treated with DNase, and total RNA was purified using the RNeasy Midi kit® (75144; QIAGEN). RNA quality was analyzed using a Bioanalyzer (Agilent), and gene expression was analyzed by the 3D-Gene platform (TORAY, Japan). As Cdx1 expression in DLD1-TetOff-Cdx1 cells was induced faster than Cdx2 in DLD1-TetOff-Cdx2 cells, RNA was purified at 12 and 24 h post-induction for Cdx1 or 24 h post-induction for Cdx2. The associated GEO accession number is GSE287318.

### ChIP-sequencing analysis

To conduct ChIP-sequencing analysis of DNA bound by Cdx1 and Cdx2, samples were prepared using a SimpleChIP® Plus Enzymatic Chromatin IP Kit (9004; Cell Signaling Technology).

For our analysis, 4–5 ×10^6^ DLD1-TetOff cells were seeded onto ten 150-mm dishes, and the expression of FLAG-tagged Cdx1 or Cdx2 was induced by withdrawing DOX from the culture medium. At 20 h post-induction, the cells were fixed by adding 540 µL of formaldehyde (a final concentration, 1%; 064-03843; FUJIFILM) to 20 mL culture medium and incubated for 5 min at 25 °C. Glycine (10×, 2 mL; 077-00735; FUJIFILM) was added to the 10 dishes, which were gently rotated for 5 min at 25 °C. The cells were washed twice with 10 mL ice-cold phosphate-based saline (PBS) and scraped with 1 mL of ice-cold PBS. Cells from the 10 dishes were pooled into a 15 mL centrifuge tube and centrifuged at 3,500 rpm (2,380 ×*g*; AX-320, TOMY) for 3 min at 4 °C, after which the supernatant was then discarded.

According to the manufacturer’s instructions of the SimpleChIP® Plus Enzymatic Chromatin IP Kit (Agarose Beads; 9004; Cell Signaling Tech.), nuclei were isolated and treated with 0.5 µL micrococcal nuclease solution (MNase; Worthington Bio. Corp.) per sample at 37 °C for 50 min. The MNase digestion reaction was terminated by adding 10 µl of 0.5 M ethylenediaminetetraacetic acid (EDTA). The size of the digested DNA (approximately 150 bp) was confirmed via agarose gel electrophoresis. The samples were centrifuged at 13,000 rpm (15,300 ×*g*; MX-307, TOMY) for 1 min at 4 °C, after which the supernatant was discarded. Each cell pellet was re-suspended in 100 µL ChIP dilution buffer (150 mM NaCl, 20 mM Tris-HCl, 1% Triton X-100, and 2 mM EDTA), incubated for 10 min on ice, transferred to TPX tubes, and sonicated to disrupt the nuclear membrane at 4 °C using a Biorupter II (Sonicbio). The samples were then centrifuged at 10,000 rpm (9,100 ×*g*; MX-307, TOMY) for 10 min at 4 °C, and then each supernatant was transferred to a new tube. A 19-fold volume of the ChIP dilution buffer was added to the digested chromatin, and each mixture was incubated with anti-FLAG® M2-antibody-conjugated agarose beads (A2220; Sigma Aldrich) for 12 h at 4 °C, with rotation. The beads were washed six times with ChIP dilution buffer and treated with RNase (final concentration, 20 µg/mL; Worthington Bio. Corp.) for 1.5 h at 4 °C. To elute the FLAG-Cdx1 or FLAG-Cdx2 complexes, the beads were incubated with 200 µL ChIP dilution buffer containing the 3×FLAG peptide (F4799; Sigma Aldrich) at 37 °C for 1 h. The samples were centrifuged at 15,000 rpm (20,400 ×*g*; MX-307, TOMY) for 1 min at 4 °C, after which 180 µL of each supernatant was transferred to a new tube. A 2× elution buffer consisting of 2% sodium dodecyl sulfate (SDS) and 0.2 M NaHCO_3_ was added to the samples, and the mixtures were incubated at 65 °C overnight. DNA was purified using the standard phenol–chloroform extraction method and analyzed on the Illumina MiSeq platform (Illumina) by Hokkaido System Science. The GEO-associated accession number is GSE287500.

### DATA analysis

Statistical analyses were performed using a Student′s *t*-test in Microsoft Excel to analyze tumor numbers (Fig. 1A–C and Fig. S1B) and data generated in luciferase reporter assays (Figs. 2B, 4C, D, 5B, 6A, 7A, and Figs. S3A, S6D), qPCR assays (Figs. 2D, E, 3A, and Fig. S3B, C), ChIP-qPCR assay (Figs. 4B, E, G, 8C, and Figs. S6A–C, E, F, S10E), proliferation assays (Fig. 3B, C), and MNase assays (Fig. 4F). The tumor numbers were obtained from four to five mice of each genotype. Luciferase reporter data are presented based on from quadruplicate assays. Data from other experiments, including qPCR, ChIP-qPCR, proliferation, and MNase assays, are generated in triplicate.

### Analysis of *CDX1/2* expression in human CRC organoids

The expression of *CDX1/2* in human CRC organoid clones was analyzed using data from datasets linked to GEO accession numbers GSM2205593–GSM2205602 [48];. The expression levels of *CDX1/2* and *LGR5* in the stem cells present within normal colonic epithelium in humans have been reported previously (Supplementary Table 2 in [49]).

## Supplementary information

Supplementary Data

Supplementary Methods

Original gel images

## Data Availability

The DNA microarray and ChIP-sequencing data have been deposited in the GEO database under accession numbers GSE287318 and GSE287500, respectively. All relevant data in this study are available from corresponding author upon reasonable request.
