# Supplementary material for: CDX1 and CDX2 suppress colon cancer stemness by inhibiting β-catenin-facilitated formation of Pol II–DSIF–PAF1C complex
Source: Cell Death Dis. 2025 May 21;16(1):408. doi: 10.1038/s41419-025-07737-3 (PMC12095478; doi:10.1038/s41419-025-07737-3)
Supplement: Supplementary file 1 — Supplementary Data [file 41419_2025_7737_MOESM1_ESM.pdf]

Supplementary Data

CDX1 and CDX2 suppress colon cancer stemness by inhibiting β-catenin-facilitated formation of Pol II–DSIF–PAF1C complex

Koji Aoki[1,2,*], Akari Nitta[1] and Ayumi Igarashi[1]
[1] Department of Pharmacology, Faculty of Medicine, University of Fukui, Fukui, Japan
[2] Life Science Support Center, University of Fukui, Fukui, Japan

[*] **Correspondence**:
Koji Aoki
Department of Pharmacology, Faculty of Medicine, University of Fukui, 23-3, Matsuokashimoaizuki, Eiheiji-cho, Yoshida-gun, Fukui 910-1193, Japan.
Phone: 81-776-61-8326; Fax: 81-776-61-8130; E-mail: aokik@u-fukui.ac.jp

Supplementary Figures 1-10
Supplementary Tables 1-3

Supplementary figure S1

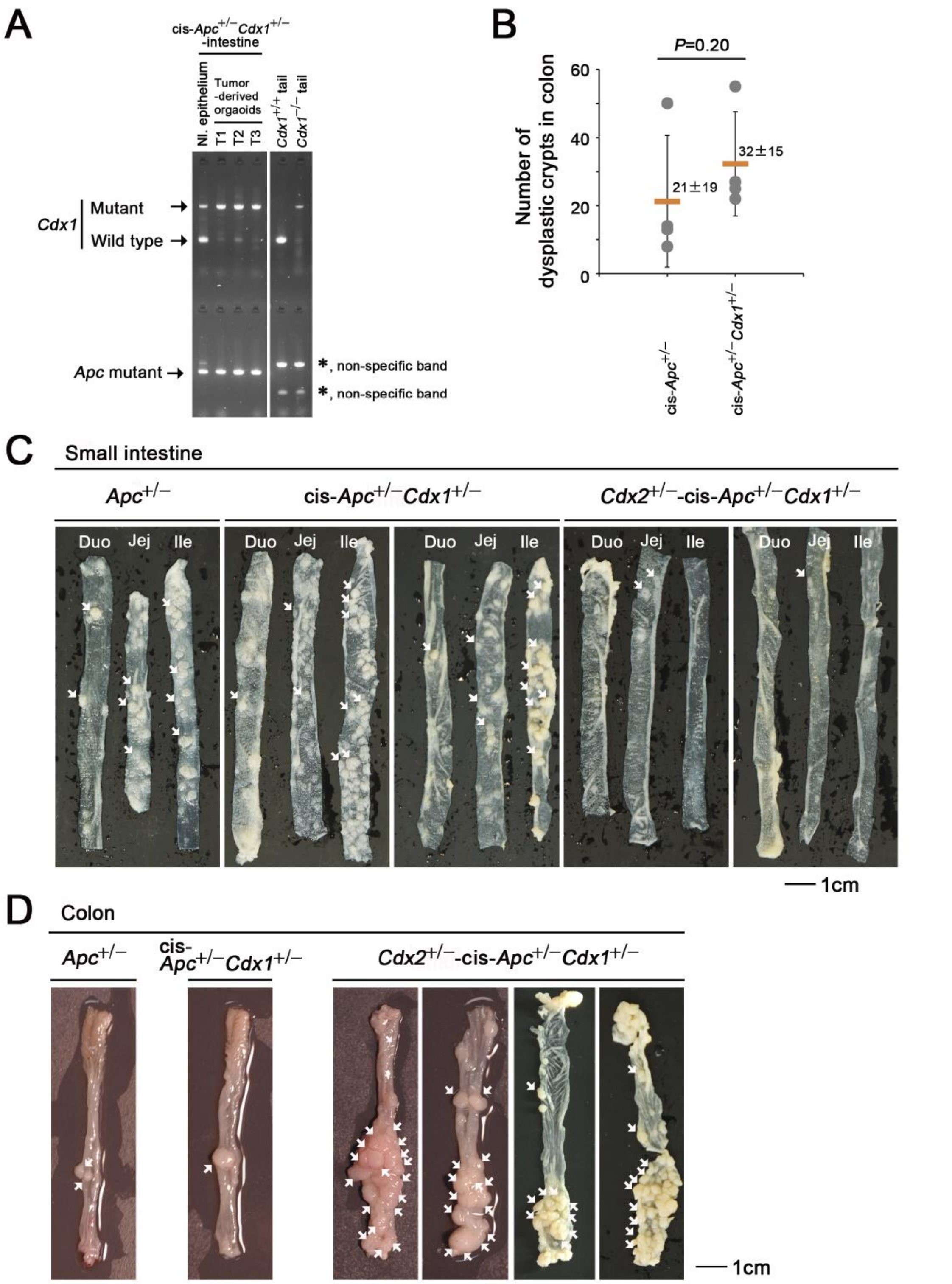

Supplementary Fig. S1: Intestinal tumors in *Apc*$^{+/-}$, cis-*Apc*$^{+/-}$*Cdx1*$^{+/-}$ and *Cdx2*$^{+/-}$-cis-*Apc*$^{+/-}$*Cdx1*$^{+/-}$ mice.

(A) Gel images of PCR-amplified wild-type and mutant *Cdx1* and *Apc* genes in

organoid cells derived from the colonic tumors of cis-*Apc*$^{+/-}$*Cdx1*$^{+/-}$ mice. DNA extracted from the tails of *Cdx1*$^{+/+}$ and *Cdx1*$^{-/-}$ mice was used as positive control for PCR analysis. The asterisk denotes non-specific bands.

(B) The number of dysplastic crypts in *Apc*$^{+/-}$ and cis-*Apc*$^{+/-}$*Cdx1*$^{+/-}$ mouse colons at 10 weeks of age (mean ± SD, n = 4). *P*-value was calculated using a Student′s *t*-test.

(C and D) Dissection micrographs of small intestines (**C**) and colons (**D**) from *Apc*$^{+/-}$ (left), cis-*Apc*$^{+/-}$*Cdx1*$^{+/-}$ (center), and *Cdx2*$^{+/-}$-cis-*Apc*$^{+/-}$*Cdx1*$^{+/-}$ (right) mice at 10–12 weeks of age. The white arrows indicate large tumors. Scale bars, 1 cm.

Supplementary figure S2

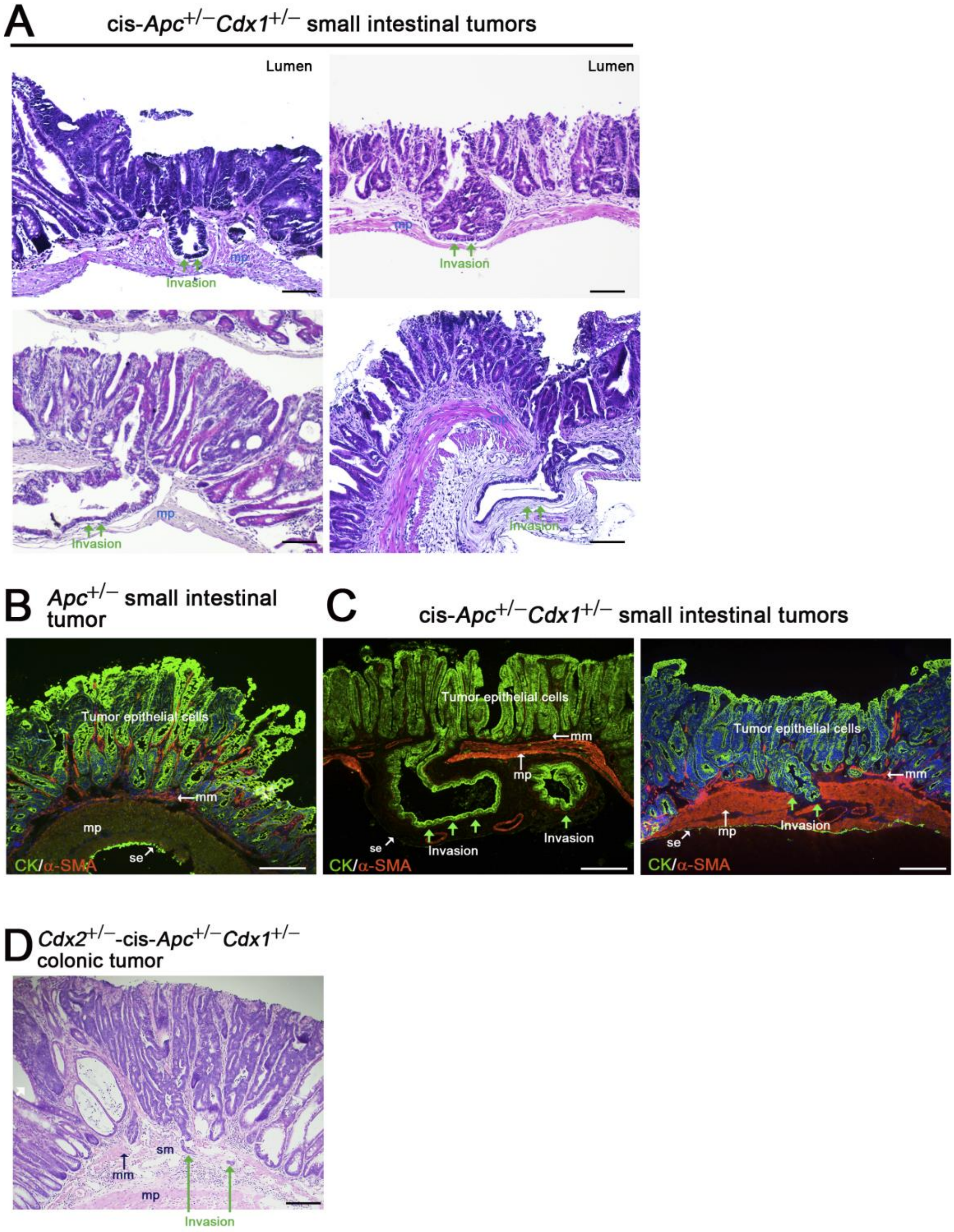

Supplementary Fig. S2: Suppression of malignant progression of colonic tumorigenesis by Cdx1 and Cdx2.

(A) Optical micrographs showing H&E-stained small intestinal tumors in cis-*Apc*$^{+/–}$*Cdx1*$^{+/–}$ mice. Green arrows indicate invasive tumor cells (**A**, **C**, **D**).

Abbreviations in **A**–**D**: mm, muscularis mucosa; mp, muscularis propria; se, serosa. The polyps were also stained with DAPI (blue). Scale bars, 200 µm (**A**–**D**).

(B and C) Fluorescence micrographs of cytokeratin (CK: green) and α-smooth muscle actin (α-SMA: red) present in small intestinal tumors in *Apc*$^{+/-}$ (**B**) and cis-*Apc*$^{+/-}$*Cdx1* mice (**C**).

(D) Optical micrographs showing an H&E-stained colonic tumor from a *Cdx2*$^{+/-}$-cis-*Apc*$^{+/-}$*Cdx1*$^{+/-}$ mouse.

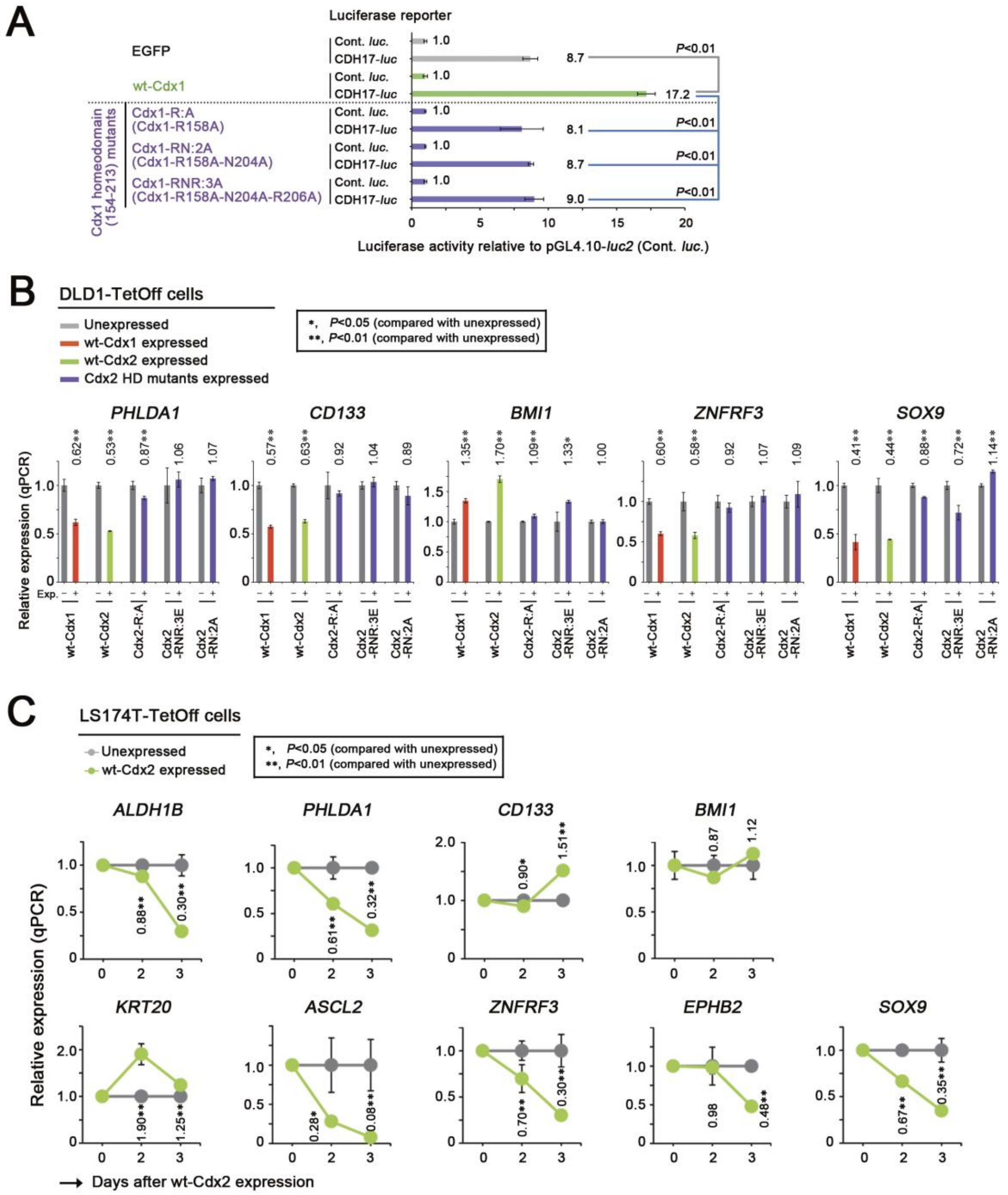

Supplementary Fig. S3: Suppression of colon cancer cell stemness by CDX1 and CDX2.

(A) Bar graph showing luciferase activities (mean ± SD) of the *CDH17 luc* reporter relative to those of the pGL4.10-*luc2* control upon expressing wt-Cdx1 or its homeodomain (HD) mutants. *P*-values were calculated using a Student′s *t*-test (**A**–**C**).

(B and **C)** qPCR data showing the relative expression (mean ± SD) of colon cancer stemness- or differentiation-related marker genes upon expression of wt-Cdx1 for 1 day or wt-Cdx2 or its homeodomain (HD) mutants for 2 days in DLD1-TetOff cells (**B**) and for 4 and 7 days in LS174T-TetOff cells (**C**), when compared with those in cells without their expression.

Supplementary figure S4

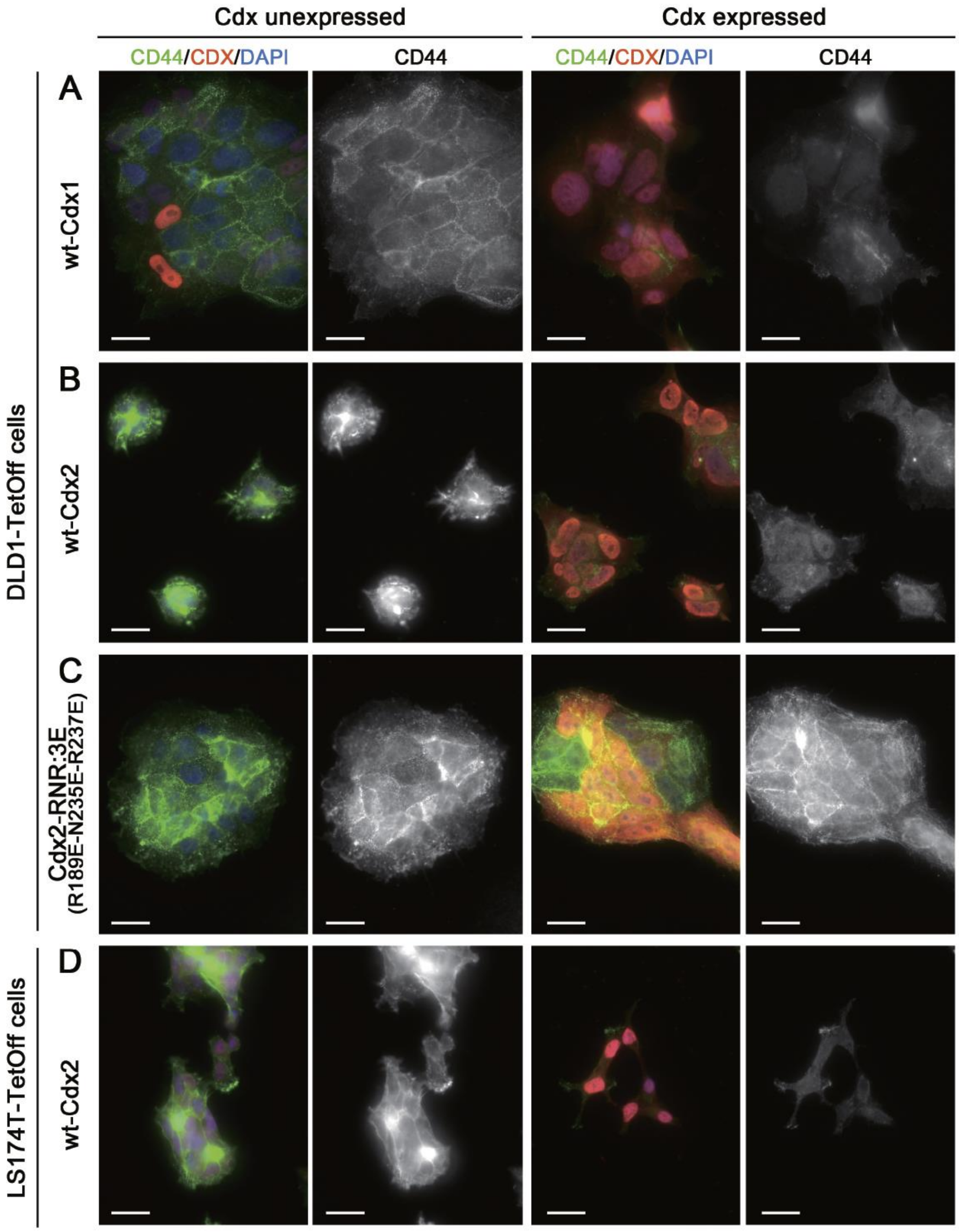

Supplementary Fig. S4: Suppression of CD44 expression by CDX1 and CDX2.

(A–D) Immunocytochemistry showing the expression of CD44 (green) and Cdx1/2 (red) upon expressing wt-Cdx1 (**A**), wt-Cdx2 (**B**, **D**), or Cdx2-RNR:3E

(**C**) for 2 days in DLD1-TetOff cells (**A**–**C**) and LS174T-TetOff cells (**D**). The cells were also stained with DAPI (blue).

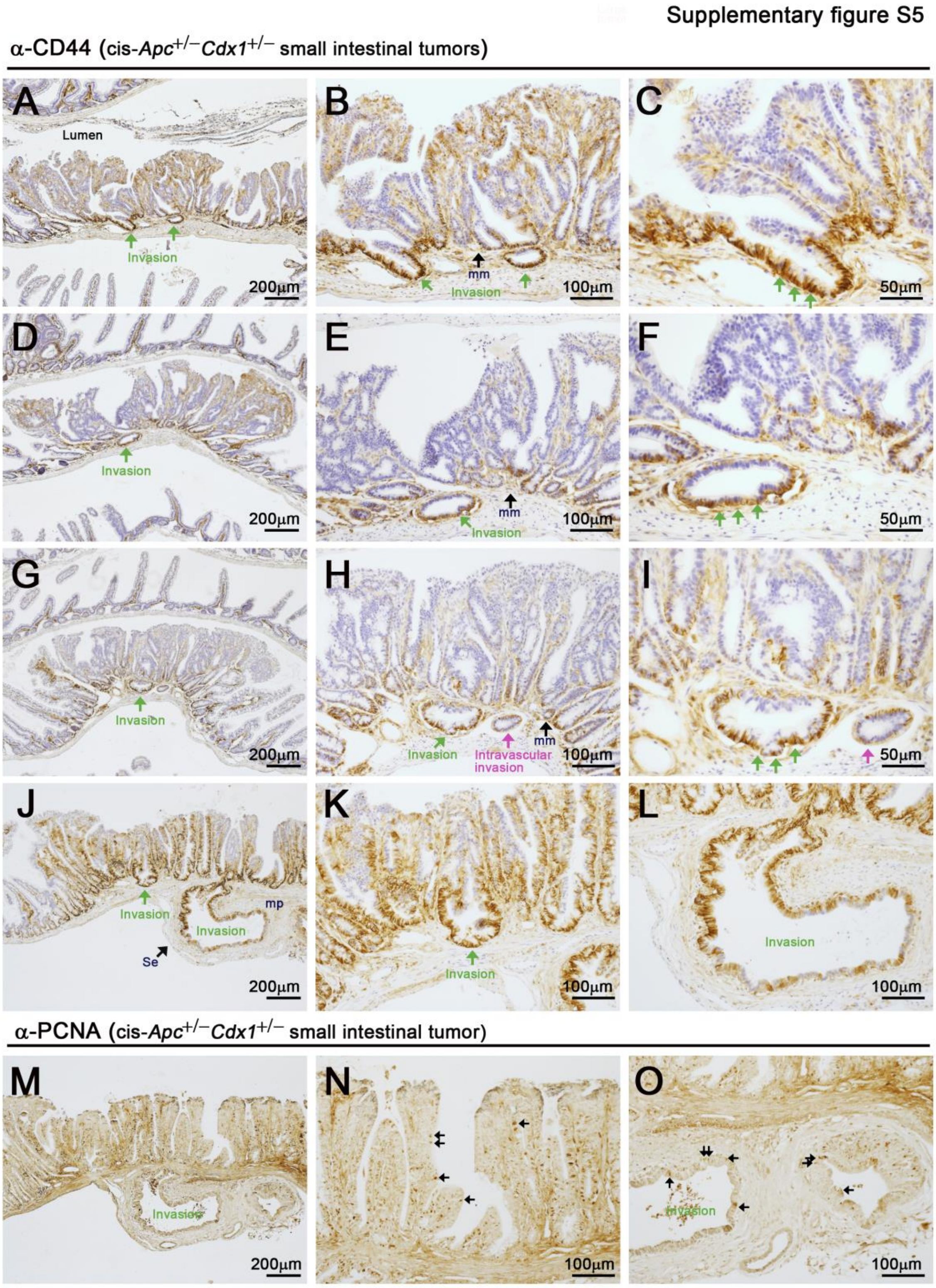

Supplementary Fig. S5: Increased Cd44 expression in the leading cells of intestinal tumor invasions in cis-*Apc*$^{+/-}$*Cdx1*$^{+/-}$ mice.

(A–I) Immunohistochemistry showing Cd44 expression (brown) in small-intestinal tumor invasions from cis-*Apc*$^{+/-}$*Cdx1*$^{+/-}$ mice. Panels (**B**), (**E**), and (**H**) are magnified images of (**A**), (**D**), and (**G**), respectively, and (**C**), (**F**), and (**I**) are magnified images of (**B**), (**E**), and (**H**), respectively. The green arrows (**C**, **F**, **I**) indicate cells with higher Cd44 expression. The tumors were counterstained with hematoxylin (blue).
(J–L) Immunohistochemistry showing Cd44 expression (brown) in a small intestinal tumor with deep invasions from cis-*Apc*$^{+/-}$*Cdx1*$^{+/-}$ mouse. Panels (**K**) and (**L**) are magnified images of (**J**). Green arrows indicate cells with higher Cd44 expression. The tumors were also counterstained with hematoxylin (blue).
(M–O) Immunohistochemistry showing PCNA expression (brown) in a small intestinal tumor with deep invasions from the cis-*Apc*$^{+/-}$*Cdx1*$^{+/-}$ mouse. Panels (**N**) and (**O**) are magnified images of (**M**). Arrows indicate cells with higher PCNA expression.

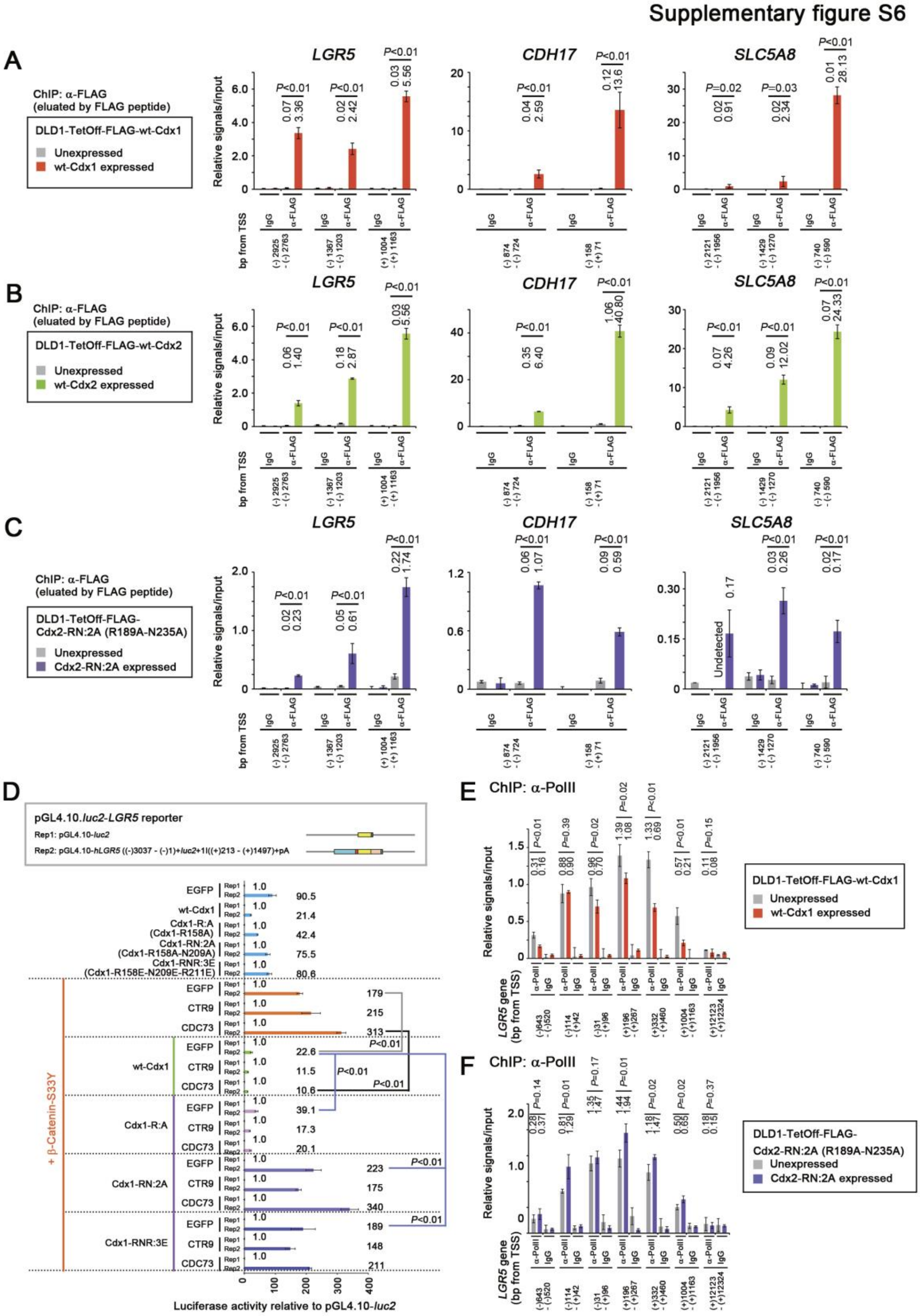

Supplementary Fig. S6: Reduced occupancy of Pol II and PAF1 on *LGR5* upon expression of CDX1 and CDX2.

(A–C) Bar graphs of ChIP-qPCR data showing the relative occupancy (mean ± SD) of exogenously expressed FLAG-tagged (**A**) wt-Cdx1, (**B**) wt-Cdx2, and (**C**) Cdx2-RN:2A (Cdx2-R189A-N235A) at the indicated positions of the *LGR5* (left), *CDH17* (center), and *SLC5A8* (right) in DLD1-TetOff cells. CDX2-target genes *CDH17* and *SLC5A8* were used as positive controls. *P*-values were calculated using a Student′s *t*-test (**A–F**).
(D) Bar graph showing the luciferase activities (mean ± SD) of the *LGR5 luc* reporter relative to those of the pGL4.10-*luc*2 control upon expressing wt-Cdx1 or its HD mutants in 293T cells. Luciferase activity was determined 40 h after transfecting the *luc* reporter into the cells.
(E and **F)** Bar graphs of ChIP-qPCR data showing the relative occupancy (mean ± SD) of Pol II at the indicated positions of *LGR5* upon expressing (**E**) wt-Cdx1 or (**F**) Cdx2-RN:2A (Cdx2-R189A-N235A) in DLD1-TetOff cells for 1 day.

Supplementary figure S7

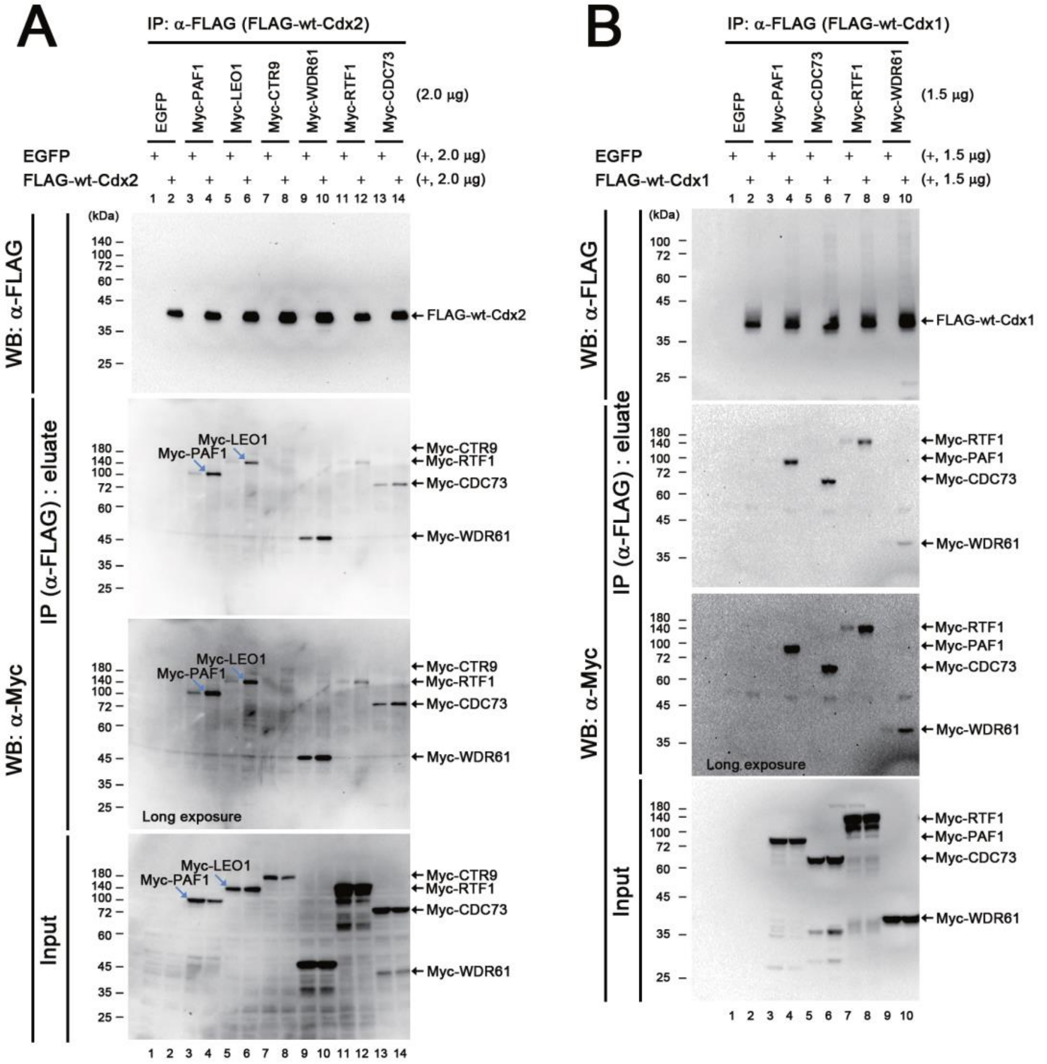

Supplemental Fig S7: Suppression of the Pol II–DSIF–PAF1C complex formation by CDX1 and CDX2.

(A and **B)** Immunoblots of IP samples showing interactions of FLAG-tagged wt-Cdx2 (**A**) or wt-Cdx1 (**B**) with Myc-tagged PAF1C components and SPT5. After IP of FLAG-wt-Cdx2 (**A**) or FLAG-wt-Cdx1 (**B**), the co-immunoprecipitated Myc-tagged proteins were analyzed via immunoblotting. The amounts of plasmid DNA transfected are indicated on the right, and long exposure gel images are shown.

Supplementary figure S8

A

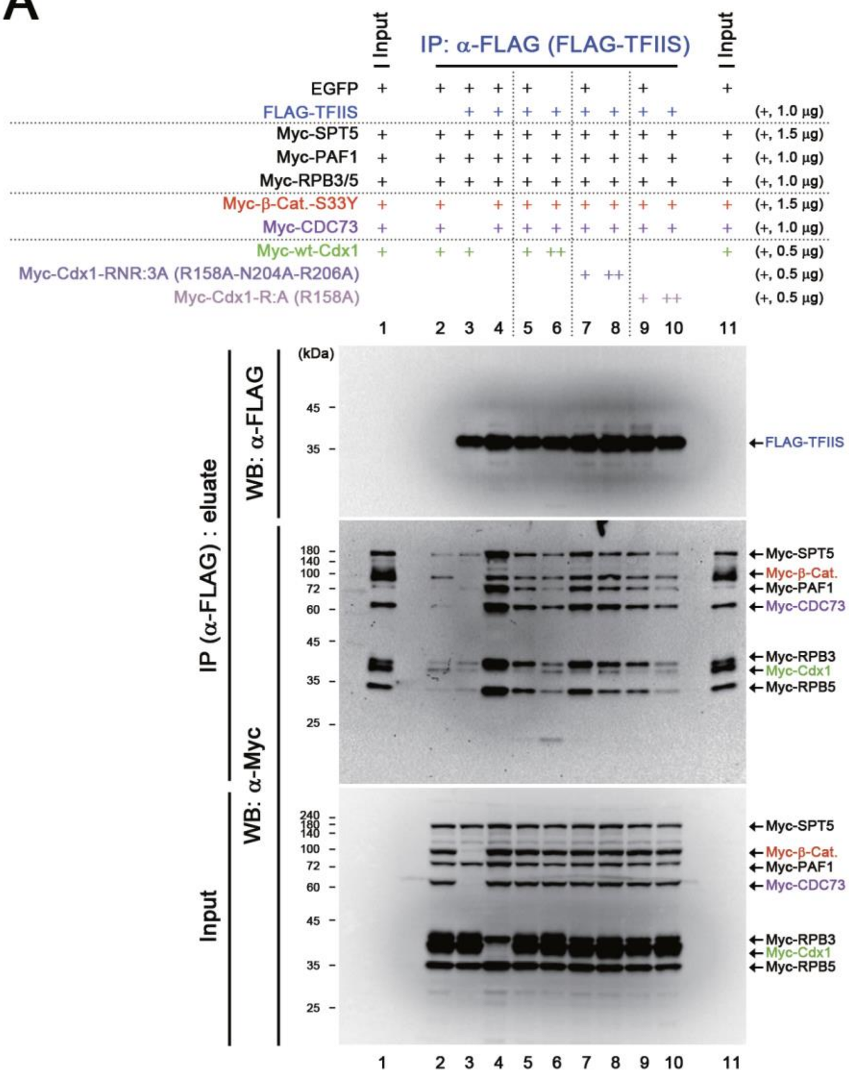

Supplementary Fig. S8: Suppression of Pol II–DSIF–PAF1C complex formation by CDX1 via its homeodomain.

(A) Immunoblots of IP samples showing the effects of wt-Cdx1 and its homeodomain (HD) mutant expression on β-catenin-S33Y (β-Cat.)-facilitated formation of a complex involving TFIIS, SPT5, PAF1, and RPBs. After IP of FLAG-TFIIS, the co-immunoprecipitated Myc-tagged proteins were analyzed via immunoblotting. The amounts of plasmid DNA transfected are indicated on the right.

Supplementary figure S9

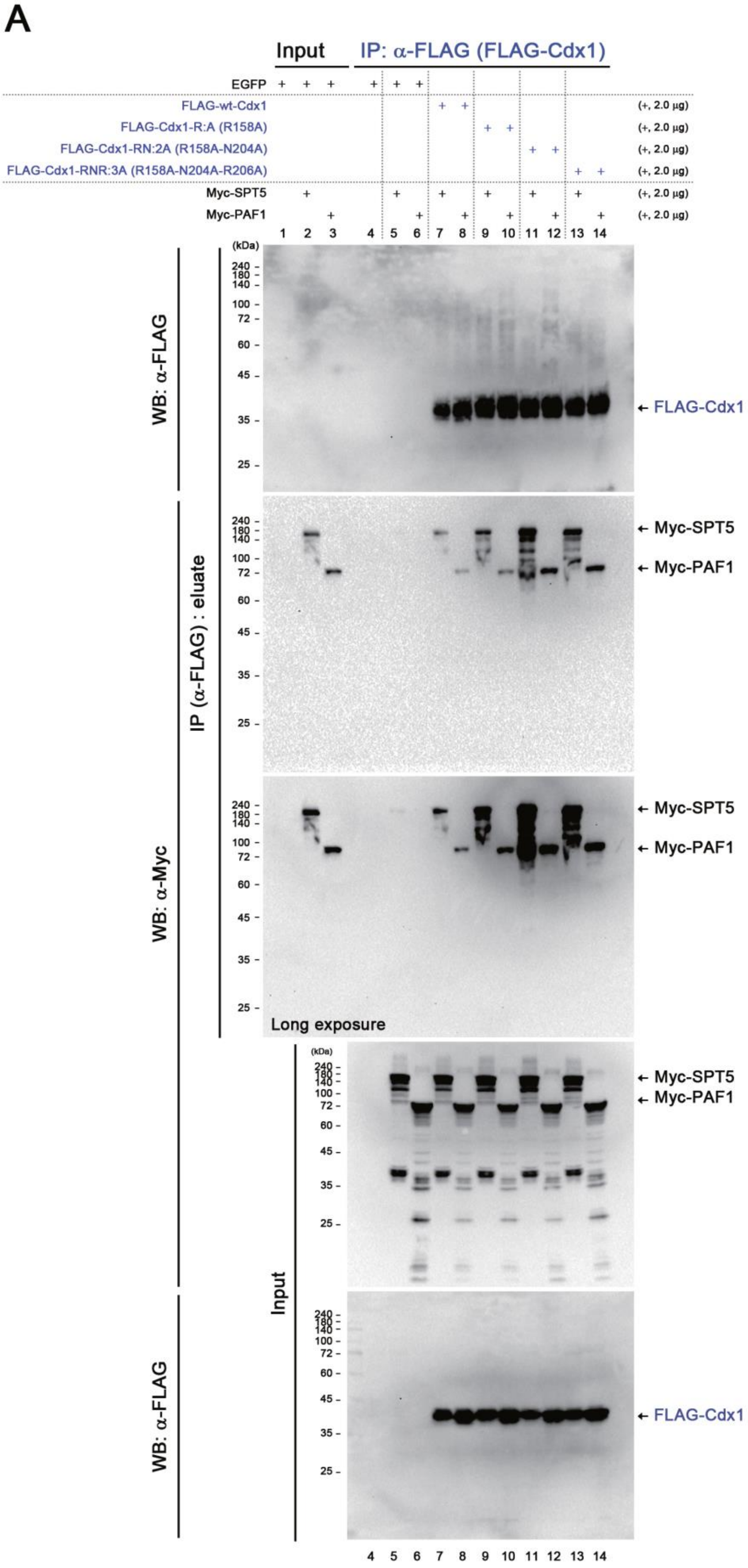

Supplementary Fig. S9: Contribution of the homeodomain to the suppression of Pol II–DSIF–PAF1C complex formation by CDX1.

(A) Immunoblots of IP samples showing interactions between SPT5 and Cdx1 (or its mutants) and between PAF1 and Cdx1 (or its mutants). After IP with FLAG-Cdx1, the co-immunoprecipitated Myc-tagged proteins were analyzed via immunoblotting. The amounts of plasmid DNA transfected are indicated on the right, and a long-exposure gel image is also shown.

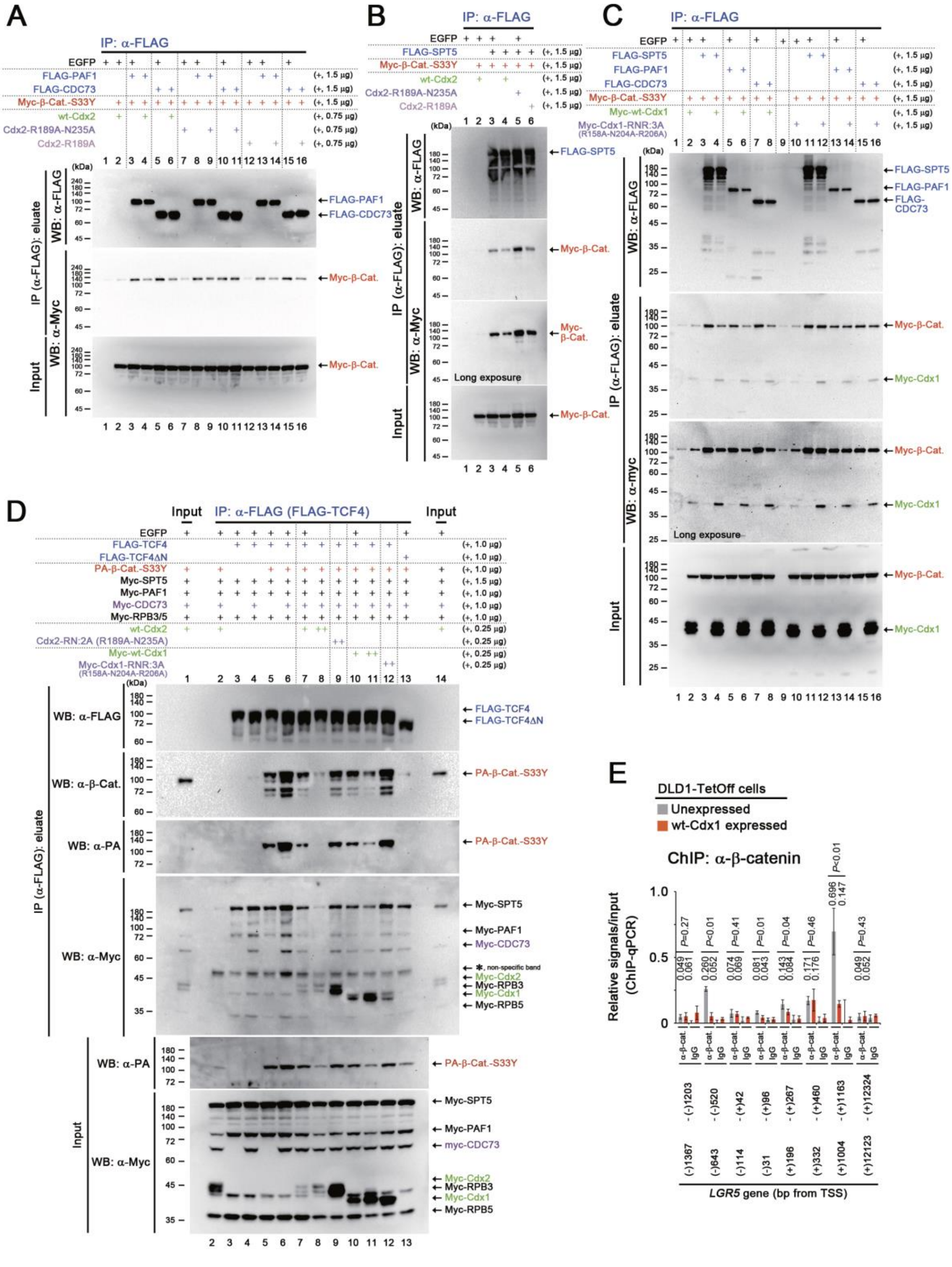

Supplementary Fig. S10: Suppression of the interaction between β-catenin and PAF1C by CDX1 and CDX2.

(A and **B)** Immunoblots of IP samples showing the effects of Cdx2 and its

homeodomain (HD) mutant expression on interactions between β-catenin-S33Y (β-Cat.) and (**A**) PAF1, CDC73, or (**B**) SPT5. After IP with (**A**) FLAG-PAF1, FLAG-CDC73, or (**B**) FLAG-SPT5, co-immunoprecipitated Myc-tagged β-catenin-S33Y was analyzed via immunoblotting. The amounts of plasmid DNA transfected are indicated on the right, and a long-exposure gel image is shown.

(C) Immunoblots of IP samples showing the effects of Cdx1 and its homeodomain (HD) mutant expression on interactions between β-catenin-S33Y (β-Cat.) and SPT5, PAF1 or CDC73. After IP with FLAG-SPT5, FLAG-PAF1, or FLAG-CDC73, the co-immunoprecipitated Myc-tagged β-catenin-S33Y was analyzed via immunoblotting. The amounts of the plasmid DNA transfected are indicated on the right, and a long-exposure gel image is shown.

(D) Immunoblots of IP samples showing the effects of wt-Cdx1/2 and their homeodomain (HD) mutant expression on the interaction of FLAG-tagged TCF4 with PA-tagged β-catenin-S33Y (β-Cat.), Myc-tagged SPT5, PAF1 components, and RPBs. Plasmid DNA expressing the indicated proteins were transfected into 293T cells, which were harvested on Day 2 for IP assay with FLAG-TCF4. The co-immunoprecipitated Myc-tagged proteins were analyzed via immunoblotting. The amounts of plasmid DNA transfected are indicated on the right. The asterisk denotes non-specific bands.

(E) Bar graph of ChIP-qPCR data showing the relative occupancy (mean ± SD) of β-catenin at the indicated positions of *LGR5* after expressing wt-Cdx1 in DLD1-TetOff cells for 1 day. IgG was used as a negative control. *P*-values were calculated using a Student′s *t*-test.

Supplementary Table S1

Summary of intestinal tumor invasion in cis-*Apc*$^{+/-}$*Cdx1*$^{+/-}$ and *Cdx2*$^{+/-}$-cis-*Apc*$^{+/-}$*Cdx1*$^{+/-}$ mice

Mouse information			Small intestinal tumors			Colonic tumors
			Depth of invasion			
Genotype	Age (weeks)	Number of mice analyzed	Submucosa	Muscularis propria	Subserosa	Invasion
cis-*Apc*$^{+/-}$*Cdx1*$^{+/-}$	12	1	+	–	–	–
	14	1	+	+	+	–
	15	2	+	+	+	–
	15	1	+	+	–	–
	16	2	+	+	+	–
	17	4	+	+	+	–
	18	2	+	+	–	–
	18	1	+	+	+	–
	19	2	+	+	+	–
	20	1	+	+	+	–
	21	1	+	+	+	–
Total	12–21	18	100% (18/18)	94% (17/18)	78% (14/18)	0% (0/18)
Cdx2$^{+/-}$-cis-*Apc*$^{+/-}$*Cdx1*$^{+/-}$	10	1	N.D.			+
	15	1	N.D.			+
	17	2	N.D.			+
	19	1	N.D.			+
	23	1	N.D.			+
Total	10–23	6	N.D.			100% (6/6)
Apc$^{+/-}$	12–20	13	0% (0/13)	0% (0/13)	0% (0/13)	0% (0/13)

N.D., not determined.
Note that development of small intestinal tumors was strongly suppressed by the *Cdx2* mutation as reported previously [10].

The depth of invasion of the small intestinal and colonic tumors is summarized for each genotype. The + sign indicates positive invasion, whereas the - sign indicates negative invasion. The number and age of the mice analyzed are also provided. The percentages of mice positive for invasion are shown at the bottom. We analyzed 18 cis-*Apc*$^{+/-}$*Cdx1*$^{+/-}$, 6 *Cdx2*$^{+/-}$-cis-*Apc*$^{+/-}$*Cdx1*$^{+/-}$ mice, and 13 *Apc*$^{+/-}$ mice.

Supplementary Table S2

The 20 genes whose expression levels were reduced the most upon inducing Cdx1 or Cdx2 in DLD1-TetOff cells

		Signal intensities									
		12 h expression of *Cdx1*			24 h expression of *Cdx1*			24 h expression of *Cdx2*			
		Unexp	Exp	Exp/ Unexp	Unexp	Exp	Exp/ Unexp	Unexp	Exp	Exp/ Unexp	
Gene number	Gene symbol	Cy5	Cy3	Cy3/ Cy5	Cy5	Cy3	Cy3/ Cy5	Cy5	Cy3	Cy3/ Cy5	Note
1	*ID1*	2974.5	376.3	0.127	2179.7	247.6	0.114	1759.3	440.4	0.250	Related to colon cancer stemness
2	*GDF15*	2790.6	377.4	0.135	3076.6	256.1	0.083	2104.9	611.4	0.290	
3	*CLDN4*	2169.8	312.1	0.144	1426.5	181.4	0.127	1705.6	505.5	0.296	
4	*TNFRSF12A*	1327.3	205.1	0.155	580.0	121.7	0.210	1034.2	363.9	0.352	
5	*DOK7*	126.7	21.0	0.166	80.7	18.9	0.234	108.9	39.4	0.362	
6	*MYC*	1025.9	170.4	0.166	673.2	84.5	0.126	443.4	102.0	0.230	Related to colon cancer stemness
7	*TNFRSF12A*	2376.9	456.0	0.192	1032.6	236.7	0.229	1772.2	820.3	0.463	
8	*RHOB*	403.7	81.2	0.201	247.7	56.7	0.229	115.3	55.8	0.485	
9	*ATAD4*	77.2	15.7	0.203	98.1	12.5	0.128	62.3	23.0	0.370	
10	*IER5L*	26.9	5.5	0.205	16.8	10.5	0.625	11.1	9.7	0.873	
11	*SLC2A1*	6059.5	1273.3	0.210	3108.9	770.3	0.248	4379.1	1470.3	0.336	
12	*RHOB*	145.5	32.3	0.222	118.9	33.2	0.279	45.9	26.7	0.582	
13	*SCNN1A*	13.0	2.9	0.222	12.6	3.7	0.297	9.6	5.3	0.558	
14	*IGFL4*	423.0	95.2	0.225	236.6	30.3	0.128	131.1	33.3	0.254	
15	*RGS16*	144.1	33.4	0.232	69.8	22.4	0.322	67.7	23.5	0.348	
16	*HOXA11*	146.9	34.1	0.232	102.7	14.6	0.142	116.0	45.8	0.395	
17	*ID3*	1279.2	300.7	0.235	844.1	215.5	0.255	738.1	294.5	0.399	Related to colon cancer stemness
18	*IER3*	1719.0	404.8	0.236	1088.1	316.8	0.291	1273.6	596.0	0.468	
19	*KIT*	263.2	64.4	0.245	287.9	46.2	0.161	127.1	46.5	0.366	
20	*EFNA1*	636.2	168.2	0.264	356.2	99.9	0.281	489.9	199.1	0.406	

The intensities of the Cy5 and Cy3 signals, along with their ratios (Cy3/Cy5), after 12 and 24 h of wt-Cdx1 expression or 24 h of wt-Cdx2 expression in DLD1-TetOff cells are shown. The genes are listed according to the most significant reduction in expression after 12 h of wt-Cdx1 expression. Abbreviations: Exp, expressed; Unexp, unexpressed.

Supplementary Table S3

Levels of *CDX1* and *CDX2* in human CRC organoids with high *LGR5* levels							
**CRC organoid 7 with knock-in of LGR5-EGFP_1**							
		**LGR5-EGFP High_1**		**LGR5-EGFP Low_1**		**Relative level**	
	GEO number	**GSM2205593**	Expression level	**GSM2205594**	Expression level	(high/low *LGR5* cells)	
Gene	Annotation	Raw value		Raw value			Average
LGR5	11733962_a_at	1.071	11.789	–1.239	0.058	204.198	
CDX1	11732512_a_at	0.112	1.294	0.143	1.391	0.931	1.022
CDX1	11732513_a_at	0.157	1.435	0.110	1.288	1.114	
CDX2	11731754_at	0.124	1.332	0.326	2.118	0.629	
**CRC organoid 7 with knock-in of LGR5-EGFP_2**							
		**LGR5-EGFP High_1**		**LGR5-EGFP Low_1**		**Relative level**	
	GEO number	**GSM2205595**	Expression level	**GSM2205596**	Expression level	(high/low *LGR5* cells)	
Gene	Annotation	Raw value		Raw value			Average
LGR5	11733962_a_at	1.221	16.616	0.370	2.343	7.091	
CDX1	11732512_a_at	0.604	4.019	0.761	5.771	0.696	0.753
CDX1	11732513_a_at	0.844	6.981	0.935	8.614	0.810	
CDX2	11731754_at	0.776	5.970	0.768	5.858	1.019	
**CRC organoid 12 with knock-in of LGR5-EGFP_1**							
		**LGR5-EGFP High_1**		**LGR5-EGFP Low_1**		**Relative level**	
	GEO number	**GSM2205597**	Expression level	**GSM2205598**	Expression level	(high/low *LGR5* cells)	
Gene	Annotation	Raw value		Raw value			Average
LGR5	11733962_a_at	0.722	5.269	–0.976	0.106	49.840	
CDX1	11732512_a_at	–1.006	0.099	–0.592	0.256	0.385	0.517
CDX1	11732513_a_at	–0.677	0.210	–0.489	0.324	0.649	
CDX2	11731754_at	–0.437	0.365	–0.396	0.402	0.909	
**CRC organoid 12 with knock-in of LGR5-EGFP_2**							
		**LGR5-EGFP High_1**		**LGR5-EGFP Low_1**		**Relative level**	
	GEO number	**GSM2205599**	Expression level	**GSM2205600**	Expression level	(high/low *LGR5* cells)	
Gene	Annotation	Raw value		Raw value			Average
LGR5	11733962_a_at	0.981	9.572	–1.693	0.020	472.368	
CDX1	11732512_a_at	–0.844	0.143	–0.669	0.214	0.668	0.595
CDX1	11732513_a_at	–0.714	0.193	–0.433	0.369	0.523	
CDX2	11731754_at	–0.108	0.781	0.108	1.281	0.609	
**CRC organoid 28 with knock-in of LGR5-EGFP_1**							
		**LGR5-EGFP High_1**		**LGR5-EGFP Low_1**		**Relative level**	
	GEO number	**GSM2205601**	Expression level	**GSM2205602**	Expression level	(high/low *LGR5* cells)	
Gene	Annotation	Raw value		Raw value			Average
LGR5	11733962_a_at	0.495	3.123	–1.323	0.048	65.679	
CDX1	11732512_a_at	–0.112	0.773	–0.148	0.712	1.085	1.182
CDX1	11732513_a_at	–0.110	0.776	–0.217	0.607	1.280	
CDX2	11731754_at	–0.332	0.466	–0.228	0.592	0.787	

CDX1 and *CDX2* expression levels were analyzed in human CRC organoids with high and low *LGR5* expression levels, using expression data deposited in GEO (accession numbers GSM2205593–GSM2205602) reported by Shimokawa *et al., Nature* 2017 **545** 187-194 (Ref. 48).
