# Supplementary material for: CDX1 and CDX2 suppress colon cancer stemness by inhibiting β-catenin-facilitated formation of Pol II–DSIF–PAF1C complex
Source: Cell Death Dis. 2025 May 21;16(1):408. doi: 10.1038/s41419-025-07737-3 (PMC12095478; doi:10.1038/s41419-025-07737-3)
Supplement: Supplementary file 2 — Supplementary Methods [file 41419_2025_7737_MOESM2_ESM.docx]

Supplementary Materials and Methods

CDX1 and CDX2 suppress colon cancer stemness by inhibiting β-catenin-facilitated formation of Pol II–DSIF–PAF1C complex

Koji Aoki^1,2,*^, Akari Nitta^1^ and Ayumi Igarashi^1^

^1^ Department of Pharmacology, Faculty of Medicine, University of Fukui, Fukui, Japan

^2^ Life Science Support Center, University of Fukui, Fukui, Japan

**^*^** **Correspondence**:

Koji Aoki

Department of Pharmacology, Faculty of Medicine, University of Fukui, 23-3, Matsuokashimoaizuki, Eiheiji-cho, Yoshida-gun, Fukui 910-1193, Japan.

Phone: 81-776-61-8326; Fax: 81-776-61-8130; E-mail: aokik@u-fukui.ac.jp

**Histological analysis**

Tissue samples were fixed with 10% formalin, and the formalin-fixed, paraffin-embedded sections were stained with hematoxylin and eosin (H&E, 30082 and 32043; Muto pure chemicals) according to standard protocols. Immuno-hisotochemistry was performed as per a previously described procedure [1] using antibodies for cytokeratin 20 (ab76126; Abcam; RRID:AB_1310117), PCNA (PC10, sc-56; SantaCruze), and CD44 (IM7, eBioscience; 14-1441-82; ThermoFisher) . Optical microscope images were captured using a BX63 microscope (Olympus Life Science) with a DP73 microscope color camera (Olympus Life Science) or a Qimaging RETIGA EXi digital camera (Roper Scientific).

**Immunoblot analysis**

Immunoblot analysis was performed as described previously [1], using anti-CDX1 [1], anti-CDX2 [1], anti-CD44 (IM7, 14-1441-82; ThermoFisher), anti-LGR5 (ab75850; Abcam), anti-c-MYC (9E10, sc40; SantaCruze), anti-β-actin (010-27841; FUJIFILM), and anti-FLAG (Anti DYKDDDDK tag, 012-22384; FUJIFILM) antibodies.

**Organoid culture**

Organoids derived from the mutant mice were cultured using a previously described procedure [2]. The cDNAs of mouse *Noggin* and *R-spondin* genes were amplified via PCR using the oligonucelotide primers listed below and cloned into a pBS plasmid. The cloned *Noggin* and *R-spondin* cDNAs were inserted into pIRESpuro3 (631619; Clontech) and pIREShyg3 (631620; Clontech) plasmid vectors. The pIREShyg3-mR-spondin plasmid was transfected into L cells (CRL-2648^TM^; ATCC) using Lipofectamine LTX^TM^ reagent with Plus^TM^ reagent (15338100; ThermoFisher Scientific). These L cells were then cultured in the presence of hygromycin (100 µg/mL) for more than 2 weeks (L-R-spondin cells).

The pIRESpuro3-mNoggin plasmid was transfected into L-mR-spondin cells, using Lipofectamine LTX reagent with PlusTM reagent (15338100; ThermoFisher Scientific), which were then cultured in the presence of puromycin (1 µg/mL) for > 2 weeks. The single-cell clones were expanded (L-R-spo.-Nog. cells). To identify L-R-spo.-Nog. cell clones expressing high levels of *Noggin* and *R-spondin*, the TOPflash/FOPflash assay (Upstate Biotechnology) was performed using a Dual-Luciferase^®^ Reporter Assay System (E1910; Promega). The pGL4.75 [lhRuc/CMV] plasmid vector (E6931; Promega) was used as the reference for transfection efficiency. Luciferase activity was measured using a GloMax^®^-Multi+ detection system (Promega). The L-R-spo.-Nog. #E2 clone was selected for subsequent experiments.

Furthermore, 4 × 10^6^ cells of the L-R-spo.-Nog. #E2 cells were used to prepare conditioned media. The L-R-spo.-Nog. #E2 clone cells were spread onto a 10-cm dish containing 8 mL of the culture media. The medium used for culturing the L-R-spo.-Nog. #E2 cells was collected over five consecutive days, diluted with advanced DMEM/F-12 (12634010; ThermoFisher Scientific), supplemented with 15% fetal bovine serum (FBS), 10 mM HEPES, Glutamax, penicillin, 10 µM Y27632, and 10 µM SB431542, and used for organoid culture.

Organoids were isolated from normal and tumor epithelia of mutant mice using a previously described procedure [2].

**Proliferation assay of organoid cells**

The growth rate of the organoids was analyzed using the CellTiter-Glo^®^ 2.0 Cell Viability Assay (G9241; Promega) and the GloMax^®^-Multi+ detection system (Promega). The organoids embedded in Matrigel (354234; CORNING) were disrupted by pipetting with 400 µL of PBS. Then, 40 µL aliquots were subjected to the CellTiter-Glo^®^ 2.0 Cell Viability Assay.

**Reverse transcriptase-qPCR (RT-qPCR) analysis**

RT-qPCR analysis was conducted as per a previously described procedure [3]. The RNA was purified with a FastGene™ RNA Basic Kit (FG-80250; Nippon Genetics). The cDNA was synthesized using ReverTra Ace (TRT-101; TOYOBO) and purified using AMPure XP (A63881; Beckman Coulter) or RNAClean XP (A63987; Beckman Coulter). The concentration of the purified cDNA was determined using the QuantiFluor^®^ ssDNA system (E3190; Promega) and the GloMax^®^-Multi+ detection system (Promega). Equal amounts of cDNAs were used for qPCR assays conducted on a StepOnePlus^TM^, QuantStuido^®^ 3 or 5 (ThermoFisher Scientific) provided by University of FUKUI, Life Science Support Center. qPCR was performed with the KOD SYBER^®^ qPCR mix (QKD-201; TOYOBO) using the oligonucleotide primers listed below. All the experiments were repeated at least twice.

**ChIP-quantitative polymerase chain reaction (ChIP-qPCR) analysis**

ChIP-qPCR analysis was performed using a previously described procedure [3]. DLD1-TetOff cells were washed with PBS to remove DOX from the culture media and were seeded onto 150-mm dishes at a density of 5-7.5 x 10^6^ cells per dish. After a predetermined period (indicated in figures) following the induction for Cdx1 or Cdx2 expression, the cells were fixed in 15 mL of 1% formaldehyde in PBS for 10 min at 25 °C with gentle rotation using a temperature-controlled shaker. The fixation was quenched by adding 1.66 mL of 1.25 M glycine to the solution, followed by a 5-min incubation at 25 °C with gentle rotation in the shaker. The cells were then washed twice with 15 mL of ice-cold PBS, scraped with 1 mL of ice-cold PBS using a cell scraper, and transferred to a 1.5-mL tube. After centrifugation at 8,000 rpm (5,800 g; MX-307, TOMY) for 1 min, the supernatants were discarded, and cells were re-suspended in 950 µL of the lysis buffer (150 mM NaCl, 50 mM Tris-HCl, 10 mM EDTA, and proteinase inhibitors), followed by incubation at 10 °C to prevent SDS precipitation. The lysate was mixed via pipetting five times. SDS (1%; 105 µL) was added to the lysate and mixed carefully via pipetting. The lysate was then incubated at 10 °C for 10 min (CTU-R; Taitec) and sonicated on a Covaris Focused-ultrasonicator M220 (Covaris) to shear DNA to fragment sizes of 200-1000 base pair fragments (setting parameters; minimum, 5.0 °C, set point, 7.0 °C, maximum, 9.0 °C; peak power, 75; duty factor, 10; cycle/burst, 200; duration, 1200 s). The sample was then centrifuged at 15,000 rpm (20,400 g; MX-307, TOMY) for 15 min at 8 °C, and the supernatant was transferred to a new tube. Protein concentrations were determined using the Bradford protein assay (500-0001; BIO-RAD) and equalized across lysates, which were then diluted five-fold in the ChIP dilution buffer (150 mM NaCl, 20 mM Tris-HCl, 1% Triton X-100, 2 mM EDTA). Immunoprecipitation assays were performed using equal amounts of lysates, with aliquots of the lysates used as input DNA.

Primary antibodies listed below were added to the diluted lysates and incubated overnight at 4 °C with rotation. Blocked protein A/G beads (Protein A/G PLUS-Agarose, sc-2003; SantaCruze) were added and further incubated for 3 h at 4 °C with rotation. The beads were washed five times with the ChIP dilution buffer and once with TE buffer. Then, the beads were eluted with an elution buffer (0.5% SDS, 50 mM Tris-HCl, and 5 mM EDTA) containing an RNase. The input DNA was treated with RNase. The samples were reverse-crosslinked overnight at 65 °C, treated by proteinase K (4548995075352; FUJIFILM), and centrifugated at 15,000 rpm for 5 min at 4 °C. The supernatants were transferred into new tubes. ChIP-DNA was purified using standard phenol-chloroform (4987481307285, 038-02606, and 135-12015; FUJIFILM) extraction and analyzed by qPCR. ChIP-qPCR signals were normalized to the input DNA signals. All the experiments were performed at least twice. qPCR was performed with the KOD SYBER^®^ qPCR mix (QKD-201; TOYOBO) using the oligonucleotide primers listed below.

**Micrococcal Nuclease (MNase) assay**

To analyze chromatin architecture, the MNase assay was performed as per a previously described procedure [4]. Briefly, 2 x 10^6^ DLD1-TetOff cells were seeded onto a 100-mm dish. Twenty-four hours after the induction of wt Cdx2 expression, the cells were fixed with 1% formaldehyde (064-03843; FUJIFILM) in PBS for 10 min at 25 °C using a temperature-controlled bioshaker (BR-33FL; TAITEC). After washing the cells with ice-cold PBS, they were scraped twice with 2.5 mL of PBS and transferred to new tubes. The samples were centrifuged at 3,500 rpm (2,380 g; AX-320, TOMY) for 3 min at 4 °C. The resulting pellets were suspended in a buffer A containing 10 mM Tris-HCl (pH 7.4), 10 mM NaCl, 3 mM MgCl_2_, 0.3 M sucrose, and 0.2% NP-40, and incubated for 10 min at 4 ºC with rotation.

After further centrifugation at 15,000 rpm (20,400 g; MX-307, TOMY) for 1 min at 4 ºC, the nuclear pellets were re-suspended in buffer A supplemented with 10 mM CaCl_2_. The samples were divided into two tubes: one was treated with 20 units of an MNase (Worthington NFCP) at 37 °C for 120 min, while the other was sonicated with a Covaris Focused-ultrasonicator M220 (setting parameters; minimum, 5.0 °C, set point, 7.0 °C, maximum, 9.0 °C; peak power 75; duty factor, 10; cycle/burst, 200; duration, 600 s).

For reverse-cross linking, 2 x elution buffer was added to the beads and incubated for 12 h at 65 °C. MNase-treated DNA was purified using a standard phenol-chloroform extraction method and subjected to agarose gel electrophoresis. DNA bands corresponding to 150 bp were excised and purified using a FastGene Gel/PCR extraction kit (FG91302; Nippon Genetics). The concentration of the extracted DNA was determined and adjusted. Equal amounts of DNA were analyzed by qPCR using the primers listed below. Relative MNase protection values were determined by normalizing the qPCR signals from MNase-treated DNA to those from sonicated DNA. All the experiments were repeated at least twice.

***Luciferase* reporter assay**

The *luciferase* assay was conducted as per a previously described procedure [3]. 293T cells were seeded in 24-well plates and transfected with 0.025 μg of either the pGL4.10 [luc2] or pGL4.10-h*LGR5* *luciferase* reporter plasmid vector, along with 0.025 μg of pGL4.75 [hRuc/CMV] plasmid vector (E6931; Promega) as the control. Additionally, the cells were transfected with 0.125 μg of plasmid vectors that expressed EGFP, β-catenin-S33Y, and PAF1C components or related molecules, or Cdx1, Cdx2 or their mutants, using a PEI MAX. Transfections were performed in quadruplicate for each plasmid set across four wells of a 24-well plate. The total amount of plasmid DNA per well was standardized by adding an EGFP-expressing plasmid vector to ensure consistency. Luciferase activity was measured approximately 40 h post-transfection using the Dual-Luciferase Reporter Assay System (E1910; Promega). Each experiment was repeated at least twice to confirm its reproducibility. The pGL4.75 [hRuc/CMV] plasmid vector (E693A; Promega) served as a reference for transfection efficiency. Luciferase signals were detected using the GloMax-Multi+ detection system (Promega). Previously constructed *LGR5* and *CDH17 luciferase* reporters has been used [1, 3].

**Immunoprecipitation assay**

Immunoprecipitation assays were performed as per a previously described procedure [3]. Briefly, 8 × 10^6^ 293T cells (CRL-1573; ATCC) were plated onto a 150-mm dish one day prior to transfection. Cells were transfected with expression vectors using Polyethylenimine MAX^TM^ (MW 40,000: 24765-100; Polysciences). Subsequently, each expression plasmid vector was transfected into the 293T cells. The amounts of the plasmid vectors transfected were indicated in figures (Fig. 4C-E, 5B, 5C, 6B, 6C, 7A, and 7B, and Fig. S4A-C, S5A, S6A, and S7A-D). In each case, the total amount of plasmid vectors transfected into 293T cells was adjusted to maintain consistency with the amount of the plasmid vector expressing EGFP.

Approximately 40 h post-transfection, the cells were washed twice with 10 mL of ice-cold PBS, scraped with 1 mL of ice-cold PBS, and transferred into 1.5-mL tubes. The cells were centrifuged at 8,000 rpm (5,800 g; MX-307, TOMY) for 1 min, and the cell pellets were lysed in a buffer containing 50 mM Tris-HCl (pH 8.0), 150 mM NaCl, 5 mM MgCl_2_, 1 mM EDTA (pH 8.0), 0.5% NP-40 and, protease and phosphatase inhibitors. The sample was incubated on ice for 15 min and then centrifuged at 15,000 rpm (20,400 g; MX-307, TOMY) for 15 min at 4 °C. The supernatant was transferred into a new tube, and the protein concentrations were determined using the Bradford protein assay. Equal amounts of protein lysates were incubated with anti-DYKDDDDK tag antibody beads (018-22783; FUJIFILM) for 3 h at 4 °C with rotation. The agarose beads were washed five times with wash buffer containing 50 mM Tris-HCl (pH 8.0), 150 mM NaCl, 0.1% NP-40, and flag-tagged protein complexes were eluted by the addition of a 3 x FLAG peptide (F4799; Sigma Aldrich: GEN-3-FLAG; Protein Ark). The SDS loading buffer was added to the eluted complex and incubated for 5 min at 95 °C. The samples were then analyzed via immunoblotting using the anbitodies listed below. Chemiluminescent signals were detected using a Chemi-Lumi One L (07880; Nacalai tesque) and a ChemiDoc^TM^ XRS Plus system (Bio-Rad). All the experiments were repeated at least twice.

**Oligonucleotide primers used for organoid culture**

| Genes | Primer names | Sequences |
| --- | --- | --- |
| R-spondin 3 | mRpo3-AIBI-ATG | ATGCATGCACCGGTGGATCCATGCACTTGCGACTGATTTCTT |
|  | mRpo3-flag-CISI-end | GCATGCATGTCGACATCGATCTACTTATCGTCGTCATCCTTGTAATCGTGTACAGTGCTGACTGAT |
| Noggin | mNg-EI-BglII-ATG | ATGCATGCGAATTCAGATCTATGGAGCGCTGCCCCAGCCTGG |
|  | mNg-NoI-NeI-end | GCATGCATGCTAGCGCGGCCGCCTAGCAGGAACACTTACACTCG |

**Antibodies and oligonucleotide primers used for ChIP-qPCR analysis**

| **Antibodies for ChIP analysis** | | | | |
| --- | --- | --- | --- | --- |
| **Antibodies** | **Clones** | **Cat. No.** | **Suppliers** | **Applications** |
| Anti DYKDDDDK tag Antibody Beads |  | 018-22783 | FUJIFILM | ChIP, IP |
| Rabbit IgG−Agarose saline suspension |  | A2909 | Sigma-Aldrich | ChIP |
| Anti-beta-catenin antibody |  | 610154 (RRID:AB_397555) | BD Transduction | ChIP, IHC |
| ANTI-FLAG® M2 Agarose Affinity Gel |  | A2220RRID (RRID: AB_10063035) | Sigma-Aldrich | ChIP, IP |
| Goat Anti-Rabbit IgG(H+L)-HRP Conjugate |  | 1706515 (RRID:AB_11125142) | Bio-Rad | ChIP |
| Anti-Mouse IgG (whole molecule) −Agarose antibody produced in goat |  | A6531 (RRID:AB_258295) | Sigma-Aldrich | ChIP, IP |
| Anti-RNA polymerase II antibody | CTD4H8 | 05-623 (RRID:AB_309852) | Merck | ChIP |
| Normal mouse IgM |  | sc-3881 (RRID:AB_737292) | SANTA CRUZ | ChIP |
| Anti-Pol II antibody | F-12 | sc-55492 (RRID:AB_10708864) | SantaCruze Biotechnology | ChIP |
| Normal Rat IgG Whole Molecule, Purified |  | 147-09521 | FUJIFILM | ChIP |
| Anti-SPT5 antibody | D-3 | sc-133217 (RRID:AB_2196394) | SantaCruze Biotechnology | ChIP |
| Anti-PAF1/PD2 antibody - ChIP Grade |  | ab20662 (RRID:AB_2159769) | Abcam | ChIP |
| CDX2-88 |  | MU392A-UC | Biogenex | ChIP |
| Anti-acetyl histone H3 (Lys27) | MABI0309 | 308-34843 | FUJIFILM | ChIP |
| Anti-H3K4me3 mouse mAb |  | 05-1339 | Upstate | ChIP |

| **Target gene positions (TSS= +1)** | **Oligonucleotide primer names** | **Sequences** |
| --- | --- | --- |
| CDH17  (-)1699 - (-)1556 | CDH17-1699F | TGCAGGGATTTCTGCCCTTGGACTT |
|  | CDH17-1556R | TCGGGACTGCAACCCTGTTCTGATT |
| CDH17  (-)874- (-)724 | CDH17 -874 F | TCAGATGCACACCTGGAGAG |
|  | CDH17 -727 R | CTGGGAATCCACACTGAAGG |
| CDH17  (-)473 - (-)366 | CDH17-473F | GTCTTTCCTCATGTCTTCTGAGTC |
|  | CDH17-366R | TCAAACACAAAAGACGGAGCA |
| CDH17  (-)158 - (+)71 | CDH17 -158F | GATACCCAGTGGCTCTCGAA |
|  | CDH17 +71R | CCTTTTCTTCCATTCAGTGGTC |
| SLC5A8  (-)2121 - (-)1958 | SLC5A8 -2121 F | TCAAATTGAAAGGACTCACCA |
|  | SLC5A8 -1956 R | GAACAGCTAGATTTTTGTTTCCTCA |
| SLC5A8  (-)1429 - (-)1270 | SLC5A8 -1429 F | GGCTAAGATATCATGGCTCGT |
|  | SLC5A8 -1270 R | TTCATCTTCTTTTGGACACCTAGA |
| SLC5A8  (-)740 - (-)590 | SLC5A8 -590 F | TCATATTATTTGAGTGTGATTCATGTG |
|  | SLC5A8 -740 R | CGTGTTATAGGTAGGCAAAATGTG |
| LGR5  (-)3710-(-)3561 | LGR5 -3710F | GAGAAATTTCCCAAAACAAAAGG |
|  | LGR5 -3561R | CCTTGGGAGCCAACTCCT |
| LGR5  (-)2647-(-)2491 | LGR5 -2647F | CTCCATCTGAGACCACCTCA |
|  | LGR5 -2491R | GTAATGGGCAGAGGTTGGAA |
| LGR5  (-)1367-(-)1203 | LGR5 -1367F | GTACTGATTGTGCGGAAACC |
|  | LGR5 -1203R | CAAACCTCGCTGTTTGTTCA |
| LGR5  (-)643-(-)520 | LGR5 -643F | TCACTTCGACTTCCTCACCCCGCAA |
|  | LGR5 -520R | CACTGTCTGGCTCGCTTTTGCCCT |
| LGR5  (-)114 - (+)42 | LGR5 -425F | ACTTGGGAAAGGAGGGAGGGGACAA |
|  | LGR5 -269R | TGAGCGGTGTGGAGCAGCATCT |
| LGR5  (-)31 - (+)96 | LGR5 -31F | GAATCTTCCAGGCGGAGGCTCAGT |
|  | LGR5 +96R | CCCACTTTCCCTCCACTTGTCCCCT |
| LGR5  (+)196 - (+)267 | LGR5 -115F | AGACGCCCGCTGAGTTGCAGAA |
|  | LGR5 +44R | AGCACAGGCAAGGACAGGAGCACA |
| LGR5  (+)332 - (+)460 | LGR5 +21F | TGTGCTCCTGTCCTTGCCTGTGCT |
|  | LGR5 +129R | ACCCTGAGCAACATCCTGCCGT |
| LGR5  (+)1004 - (+)1163 | LGR5 +1004F | GCATCCCTTGCACTGTGAC |
|  | LGR5 +1163R | CTAATCGGGGTTTGTGGTTG |
| LGR5 (1st intron)  (+)12123 - (+)12324 | LGR5intron1-2F | CCAAAACCCACAACCCCACAACCAC |
|  | LGR5intron1-2R | CCACGGGAAGGAATGGACAAGGCA |
| Myoglobin 1 (+) 290 - (+) 411 | hMyoglobin1-290F | GTGTTAGAACGGTCACTTGCTTTT |
|  | hMyoglobin1-411R | CTAGGTTTGATTCTCATGCTTCCTC |

**Oligonucleotide primers used for mutagenesis of *Cdx1* and *Cdx2***

| **Mutants** | **Primer names** | **Sequences** |
| --- | --- | --- |
| mCdx1-R158A | mCdx1-R158A-F | gacaagtacGAAgtggtctacacagaccaccaa |
|  | mCdx1-R158A-R | gtagaccacTTCgtacttgtccttggttcgggt |
| mCdx1-R158A-R204A | mCdx1-R158A-R204A-F | ttccagGCCcgcGCGgccaaggagcgcaaagtaaac |
|  | mCdx1-R158A-R204A-R | cttggcCGCgcgGGCctggaaccagatctttacctg |
| mCdx1-R158A-R204A-R206A | mCdx1-R158A-R204A-R206A-F | ttccagGCCcgcGCGgccaaggagcgcaaagtaaac |
|  | mCdx1-R158A-R204A-R206A-R | cttggcCGCgcgGGCctggaaccagatctttacctg |
| mCdx2-R189A | mCdx2-R189A-F | aggacaaaagacaaatacgcggtggtg |
|  | mCdx2-R189A-R | atggtctgtgtacaccaccgcgtattt |
| mCdx2-N235A | mCdx2-N235A-F | gttaaaatttggtttcaggcccgcaga |
|  | mCdx2-N235A-R | cctctccttggctctgcgggcctgaaa |
| mCdx2-R237A | mCdx2-R237A-F | atttggtttcagaaccgcgcagccaag |
|  | mCdx2-R237A-R | gattttcctctccttggctgcgcggtt |
| mCdx2-R237E | mCdx2-R237E-F | aaaatttggtttcagaaccgcgaagccaaggag |
|  | mCdx2-R237E-R | cttgattttcctctccttggcttcgcggttctg |
| mCdx2-R189E | mCdx2-R189E-F | gacaaatacgaggtggtgtacacagaccatcag |
|  | mCdx2-R189E-R | gtacaccacctcgtatttgtcttttgtcctggt |
| mCdx2-N235E | mCdx2-N235E-F | tttcaggaacgcagagccaaggagagg |
|  | mCdx2-N235E-R | tctgcgttcctgaaaccaaattttaac |
| mCdx2-N235A-R237A | mCdx2-N235A-R237A-F | tttcaggcccgcgcagccaaggagaggaaaatcaag |
|  | mCdx2-N235A-R237A-R | cttggctgcgcgggcctgaaaccaaattttaacctg |
| mCdx2-N235E-R237E | mCdx2-N235E-R237E-F | tggtttcaggaacgcgaagccaaggagaggaaaatcaag |
|  | mCdx2-N235E-R237E-R | ctccttggcttcgcgttcctgaaaccaaattttaacctg |

**Oligonucleotide primers used for RT-qPCR analysis**

| **Target genes** | **Oligonucleotide primer names** | **sequences** |
| --- | --- | --- |
| *CD44* | hCD44pv9F | CAGAGCTTCTCTACATCACA |
|  | hCD44hs3R | TTTGCTCCACCTTCTTGACTCC |
| *ID1* | ID1-189F | CGTGCTGCTCTACGACATGA |
|  | ID1-339R | GGATTCCGAGTTCAGCTCCA |
| *ID3* | ID3-46F | TGCCTGTCGGAACGCAGTCT |
|  | ID3-311R | AGCTCGGCTGTCTGGATGGGAA |
| *LGR5* | LGR5-1788F | TGGGGTCATCGCAGCAGTGA |
|  | LGR5-2167R | TGGTGCTGGGCTCCCCAAAA |
| *SOX9* | SOX9-316F | AAGCGGCCCATGAACGCCTT |
|  | SOX9-623R | TGCAGCGCCTTGAAGATGGC |
| *ASCL2* | ASCL2-180F | CAACCGCGTGAAGCTGGTGA |
|  | ASCL2-296R | ATGTACTCCACGGCTGAGCGCA |
| *ZNFRF3* | ZNFRF3-2345F | ATGAAGAGAAGCAGGTGGCC |
|  | ZNFRF3-2747R | GTGCTGGACTCCTGAGTGTC |
| *RNF43* | RNF43-1929F | AAAATCCAGCCTCTCTGCCC |
|  | RNF43-2108R | GGTCCACAGATCAAGGGGTG |
| *MYC* | Myc-468F | CAAAGACAGCGGCAGCCCGA |
|  | Myc-633R | TTGCGAGGCGCAGGACTTGG |
| *EPHB2* | EPHB2-2237F | ACCTGGCTGCCCGCAACATC |
|  | EPHB2-2487R | CCAGTAGGGCCGCTCCCCAT |
| *ALDH1B* | ALDH1B-673F | ATCAAGGAGGCAGGCTTTCC |
|  | ALDH1B-1098R | CTGCTCCTTGTCCACCTGAG |
| *BMI1* | hBMI1-37F | AATCCCCACCTGATGTGTGT |
|  | hBMI1-435R | CCGATCCAATCTGTTCTGGT |
| *KRT20* | KRT20-693F | AGGCCTGAACCTTGGCGTCAT |
|  | KRT20-971R | TGGCTGCTGTAACGGGCCTT |
| *PROM1 (CD133)* | PROM1-340F | GTCCTGGGGCTGCTGTTTAT |
|  | PROM1-472R | AGCATTTCCTCAGGAAGGGC |
| *PHLDA1* | PHLDA1-771F | AGAGGGCAAGGAGATCGACT |
|  | PHLDA1-1096R | GGTGCGAGTGAGGATGAGAG |

| **Target genes** | **Oligonucleotide primer names** | **sequences** |
| --- | --- | --- |
| *mLgr5* | mLgr5-2486F | GGATATGGGCAGCCTGGGAAAGCA |
|  | mLgr5-2658R | ATGCCCCGGAAGTGGAAGGCAA |
| *mLgr5* | mLgr5-2274F | TCTTTGGGATTGTTCGATGGTGAAG |
|  | mLgr5-2459R | TAGAGAAGTGGGTTGAGACAGGAAG |
| *mCdx1* | mCdx1-895F | AGCCTGGGGTCTAGGGATCTAGGGA |
|  | mCdx1-1056R | CAACGGAAGGCAGGAAGAGGGCAAG |
| *mCdx2* | mCdx2-24F | CAAGGACGTGAGCATGTATCC |
|  | mCdx2-129R | GTAACCACCGTAGTCCGGGTA |

**Antibodies used for immunoprecipitation assay**

| **Antibodies used for WB analysis** | | | | |
| --- | --- | --- | --- | --- |
| **Antibodies** | **Clones** | **Cat. No.** | **Suppliers** | **Applications** |
| Anti DYKDDDDK tag, Monoclonal Antibody |  | 018-22381 | FUJIFILM | WB |
| Anti-beta-catenin antibody |  | 610154 (RRID:AB_397555) | BD Transduction | ChIP |
| Anti β-Actin, Monoclonal Antibody |  | 013-24553 | FUJIFILM | WB |
| Anti-Myc-tag mAb |  | M192−3 (RRID:AB_11160947) | MBL | WB |
| Anti-PA tag, Rat monoclonal antibody |  | 012-25863 | FUJIFILM | WB |

**Oligonucleotide primers used for MNase assay**

| **Target gene positions**  **(TSS = +1)** | **Primer names** | **Sequences** |
| --- | --- | --- |
| LGR5  (-)215 - (-)77 | LGR5 -215F | GCGGCGTTGAGCACTGAATCTTCCA |
|  | LGR5 -77R | ACTTTCCCTCCACTTGTCCCCTCCC |
| LGR5  (-)98 - (+)28 | LGR5 -98F | AGGGGACAAGTGGAGGGAAAGTGGG |
|  | LGR5 +28R | AGCATCTCTGCTTGCACGCTCGTTT |
| LGR5  (-)22 - (+)43 | LGR5 -22F | GCAATTCGGGCTGGAGCGCTTT |
|  | LGR5 +43R | TGAGCGGTGTGGAGCAGCATCT |
| LGR5  (-)46 - (+)79 | LGR5 -46F | GCGGCGTTGAGCACTGAATCTTCCA |
|  | LGR5 +79R | TGTCCCCTCCCTCCTTTCCCAAGTC |
| LGR5  (-)31 - (+)96 | LGR5 -31F | GAATCTTCCAGGCGGAGGCTCAGT |
|  | LGR5 +96R | CCCACTTTCCCTCCACTTGTCCCCT |
| LGR5  (+)61 - (+)169 | LGR5 +61F | GGAAAGGAGGGAGGGGACAAGTGGA |
|  | LGR5 +169R | AAAGCGCTCCAGCCCGAATTGC |
| LGR5  (+)72 - (+)196 | LGR5 +72F | AGGGGACAAGTGGAGGGAAAGTGGG |
|  | LGR5 +196R | GCATCTCTGCTTGCACGCTCGTTTT |
| LGR5  (+)148 - (+)212 | LGR5 +148F | GCAATTCGGGCTGGAGCGCTTT |
|  | LGR5 +212R | TGAGCGGTGTGGAGCAGCATCT |
| LGR5  (+)189 - (+)305 | LGR5 +189F | AGAGATGCTGCTCCACACCGCTCA |
|  | LGR5 +305R | GGACAGAGGTGCCGGTTG |
| LGR5  (+)197 - (+)322 | LGR5 +197F | AGACGCCCGCTGAGTTGCAGAA |
|  | LGR5 +322R | GAGGTGTCCATGGTGCCCGAAGTAG |
| LGR5  (+)299 - (+)355 | LGR5 +299F | TACTTCGGGCACCATGGACACCTCC |
|  | LGR5 +355R | AGCACAGGCAAGGACAGGAGCACA |
| LGR5  (+)332 - (+)463 | LGR5 +332F | TGTGCTCCTGTCCTTGCCTGTGCT |
|  | LGR5 +463R | TCCACCCTGAGCAACATCCTGCCGT |
| LGR5  (+)366 - (+)490 | LGR5 +366F | AGACGCCCGCTGAGTTGCAGAA |
|  | LGR5 +490R | AGGTGTCCATGGTGCCCGAAGTAG |
| LGR5  (+)466 - (+)524 | LGR5 +466F | CCTACTTCGGGCACCATGGACACCT |
|  | LGR5 +524R | AGCACAGGCAAGGACAGGAGCACA |
| LGR5  (+)488 - (+)587 | LGR5 +488F | GCTGCCTTCCAACCTCAGCGTCTTC |
|  | LGR5 +587R | GCCTTGGACGAACCTTCCTTCCCCT |
| LGR5  (+)567 - (+)659 | LGR5 +567 F | GAAGGAAGGTTCGTCCAAGGCGAGG |
|  | LGR5 +659 R | TGGCCAGGATGCCCTTGACAAACTC |
| LGR5  (+)631 - (+)743 | LGR5 +631 F | GGGCGAGTTTGTCAAGGGCATCCT |
|  | LGR5 +743 R | CGAAGCCATTTCTCTGCGGGTCCA |
| LGR5  (+)801 - (+)884 | LGR5 +801 F | TCGCAGGGCGAATTCCTGCAAATG |
|  | LGR5 +884 R | AGCCTGCCTTTGGGTACCAGGCTCT |
| LGR5  (+)860 - (+)989 | LGR5 +860 F | AGAGCCTGGTACCCAAAGGCAGGCT |
|  | LGR5 +989 R | TGGAGCTGGGCAAGTTGTTTCTCCT |
| LGR5  (+)965 - (+)1027 | LGR5 +965 F | AGGAGAAACAACTTGCCCAGCTCCA |
|  | LGR5 +1027 R | TCACGTCACAGTGCAAGGGATGCAG |
| LGR5  (+)998 - (+)1088 | LGR5 +998 F | TTAGTCTGCATCCCTTGCACT |
|  | LGR5 +1088 R | AAGGAAGGAAGAAGGGAGGA |
| LGR5  (+)1054 - (+)1164 | LGR5 +1054 F | TTTTCCTCCCTTCCTTCCTC |
|  | LGR5 +1164 R | CTAATCGGGGTTTGTGGTTG |
| LGR5  (+)1136 - (+)1203 | LGR5 +1136 F | AAAATGTGGCAACCACAAACCCCGA |
|  | LGR5 +1203 R | GCGCGGGCAGCCAAAGGTAAAGATA |
| LGR5  (+)1154 - (+)1282 | LGR5 +1154 F | ACCCCGATTAGAGCTGCCAGAACG |
|  | LGR5 +1282 R | TTCAGCCCACTTCCCCTTTCCTCCC |
| LGR5  (+)1266 - (+)1332 | LGR5 +1266 F | AGGGGAAGTGGGCTGAAGGCTGTT |
|  | LGR5 +1332 R | TTCAGGTCTCCCTTTGGGTGTCGGT |

**Supplementary References**

1. Aoki K, Kakizaki F, Sakashita H, Manabe T, Aoki M, Taketo MM. Suppression of colonic polyposis by homeoprotein CDX2 through its nontranscriptional function that stabilizes p27^Kip1^. Cancer Res. 2011;71:593-602.

2. Miyoshi H, Stappenbeck TS. In vitro expansion and genetic modification of gastrointestinal stem cells in spheroid culture. Nat. Protoc. 2013;8:2471-2482.

3. Aoki K, Nitta A, Igarashi A. NELF and PAF1C complexes are core transcriptional machineries controlling colon cancer stemness. Oncogene. 2024;43:566-577.

4. Jimeno-Gonzalez S, Ceballos-Chavez M, Reyes JC. A positioned +1 nucleosome enhances promoter-proximal pausing. Nucleic Acids Res. 2015;43:3068-3078.
