# Supplementary material for: CDX1 and CDX2 suppress colon cancer stemness by inhibiting β-catenin-facilitated formation of Pol II–DSIF–PAF1C complex
Source: Cell Death Dis. 2025 May 21;16(1):408. doi: 10.1038/s41419-025-07737-3 (PMC12095478; doi:10.1038/s41419-025-07737-3)

Gel and immunoblot images in Fig S1 and Fig. 2

Fig. S1A

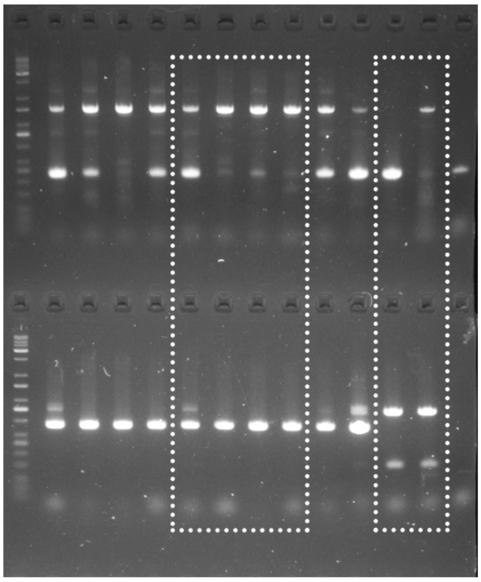

Fig. 2A

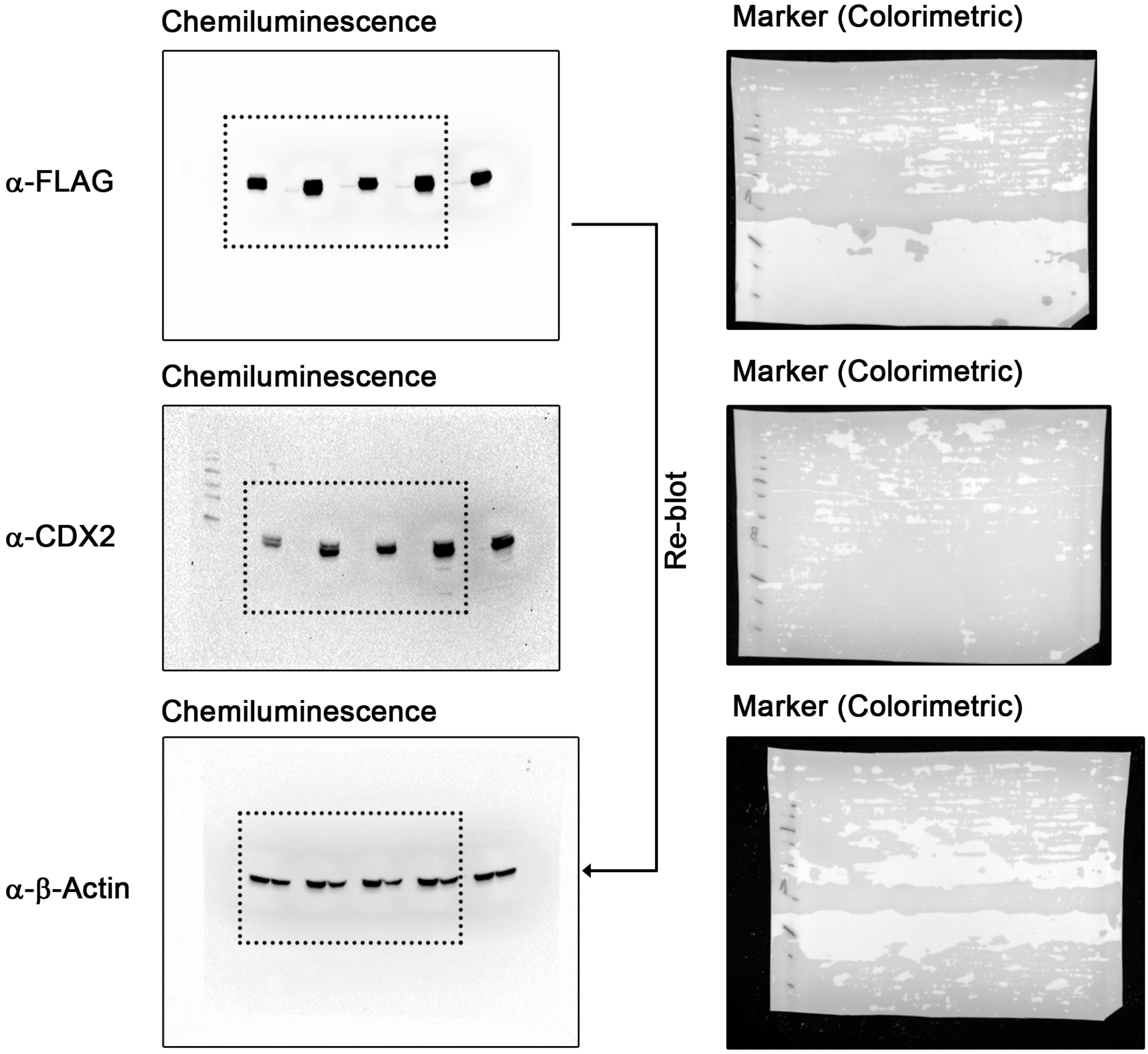

Fig. 2F

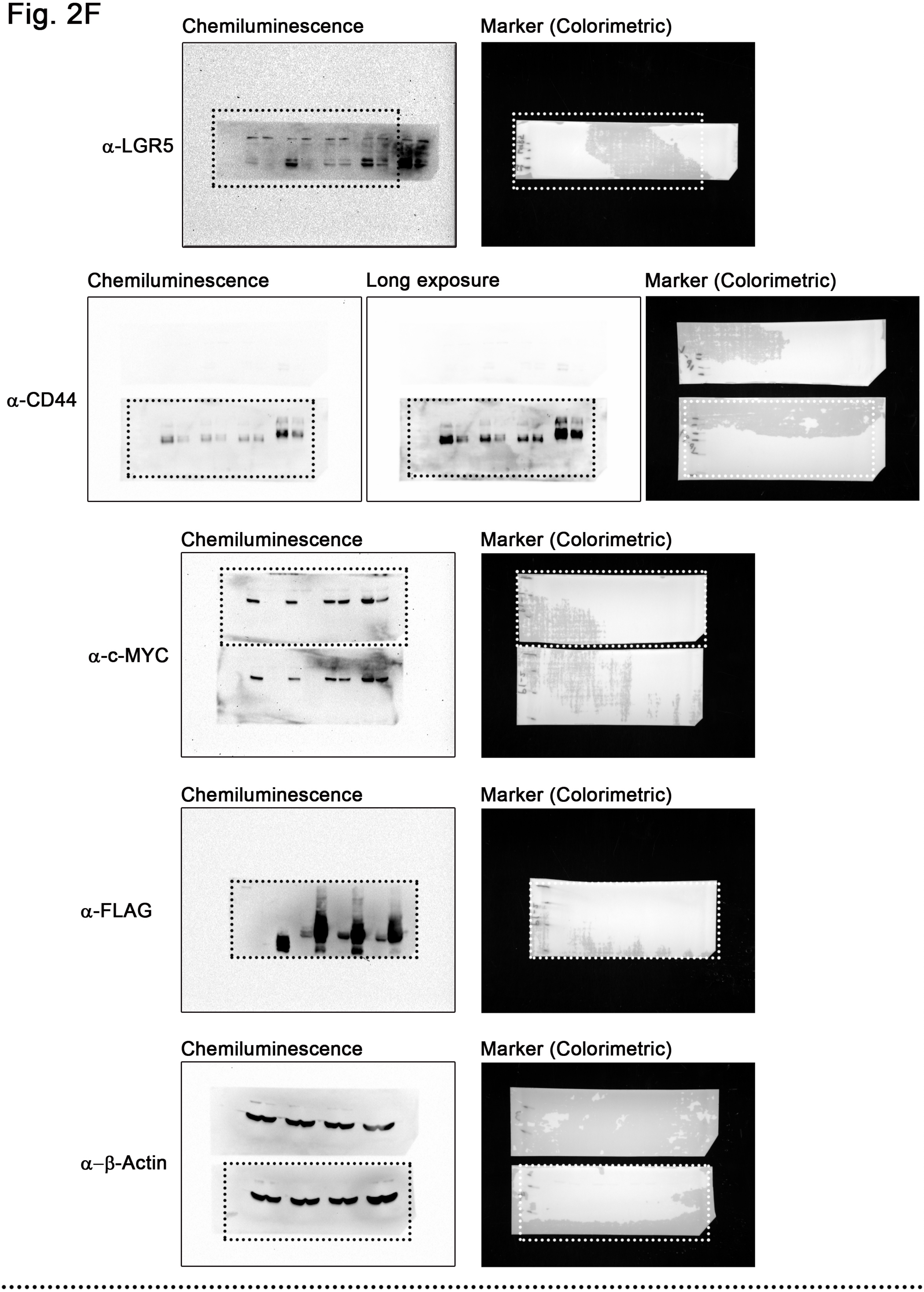
Chemiluminescence
Marker (Colorimetric)
α-LGR5
Chemiluminescence
Long exposure
Marker (Colorimetric)
α-CD44
Chemiluminescence
Marker (Colorimetric)
α-c-MYC
Chemiluminescence
Marker (Colorimetric)
α-FLAG
Chemiluminescence
Marker (Colorimetric)
α–β-Actin

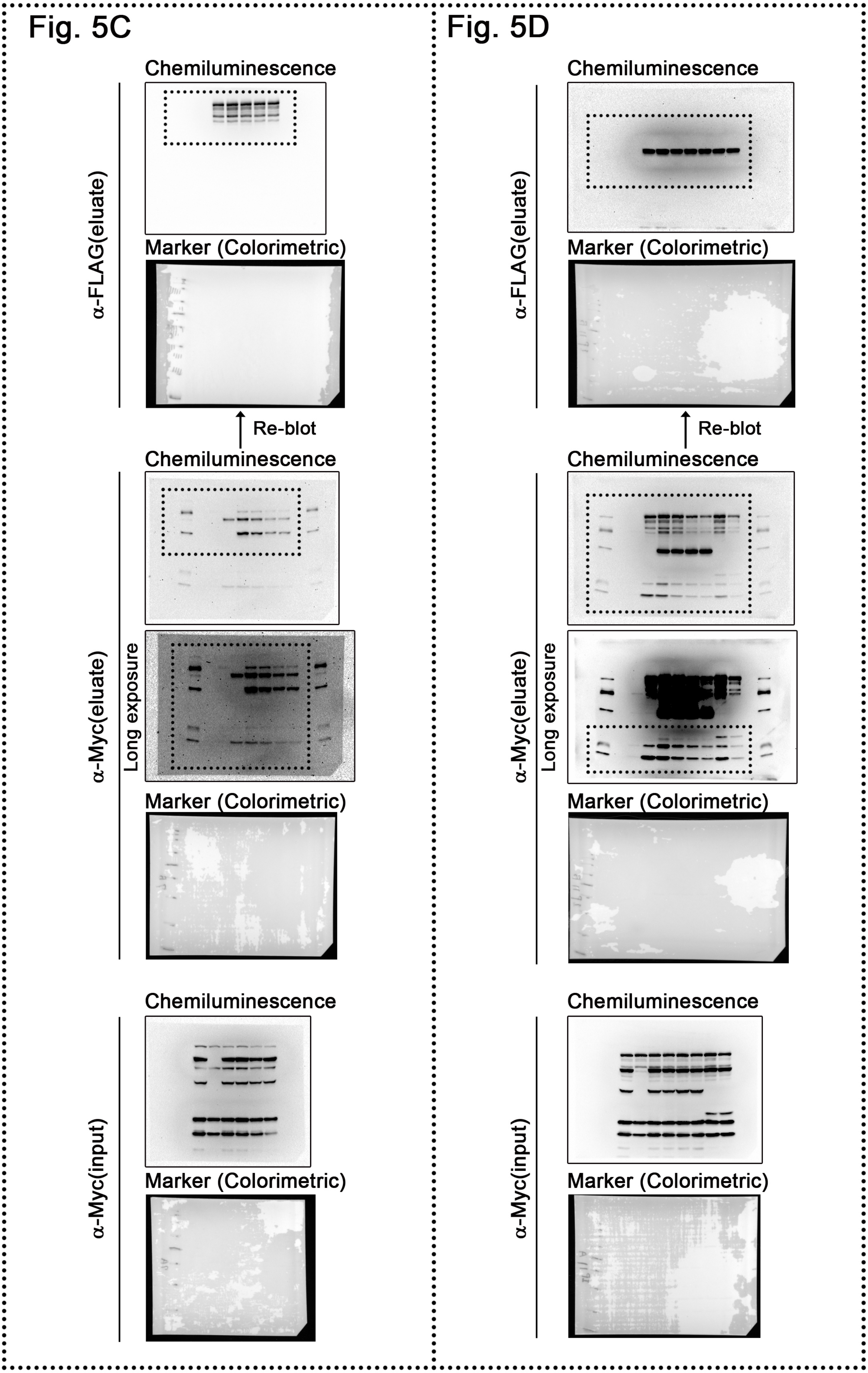
Fig. 5C
Chemiluminescence
Marker (Colorimetric)
α-FLAG(eluate)
Re-blot
Chemiluminescence
Long exposure
Marker (Colorimetric)
α-Myc(eluate)
Chemiluminescence
Marker (Colorimetric)
α-Myc(input)
Fig. 5D
Chemiluminescence
Marker (Colorimetric)
α-FLAG(eluate)
Re-blot
Chemiluminescence
Long exposure
Marker (Colorimetric)
α-Myc(eluate)
Chemiluminescence
Marker (Colorimetric)
α-Myc(input)

Fig. 5E

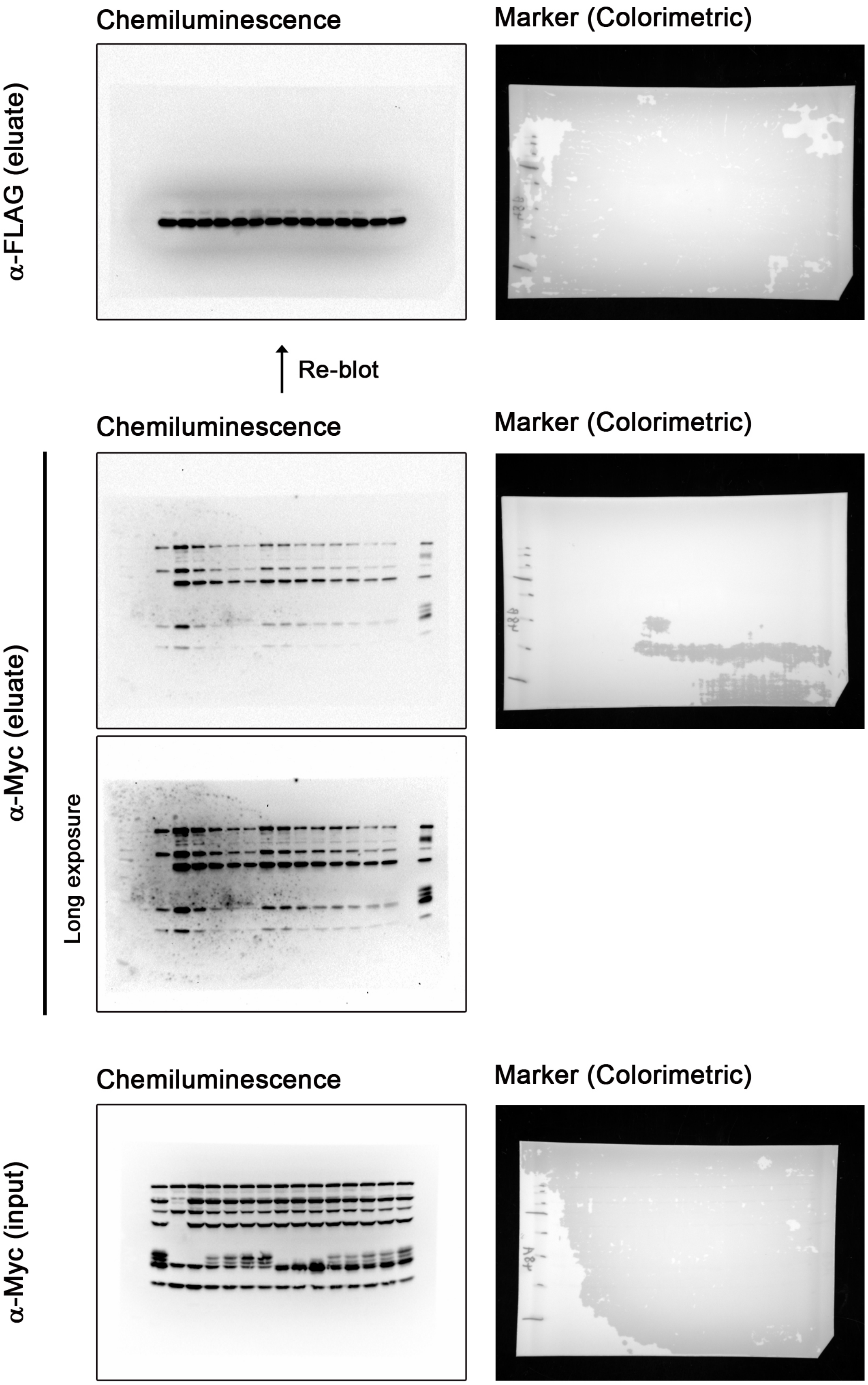

Chemiluminescence
Marker (Colorimetric)
α-FLAG (eluate)
Re-blot
Chemiluminescence
Marker (Colorimetric)
α-Myc (eluate)
Long exposure
Chemiluminescence
Marker (Colorimetric)
α-Myc (input)

Immunoblot images in Fig. 6 and Fig. S8

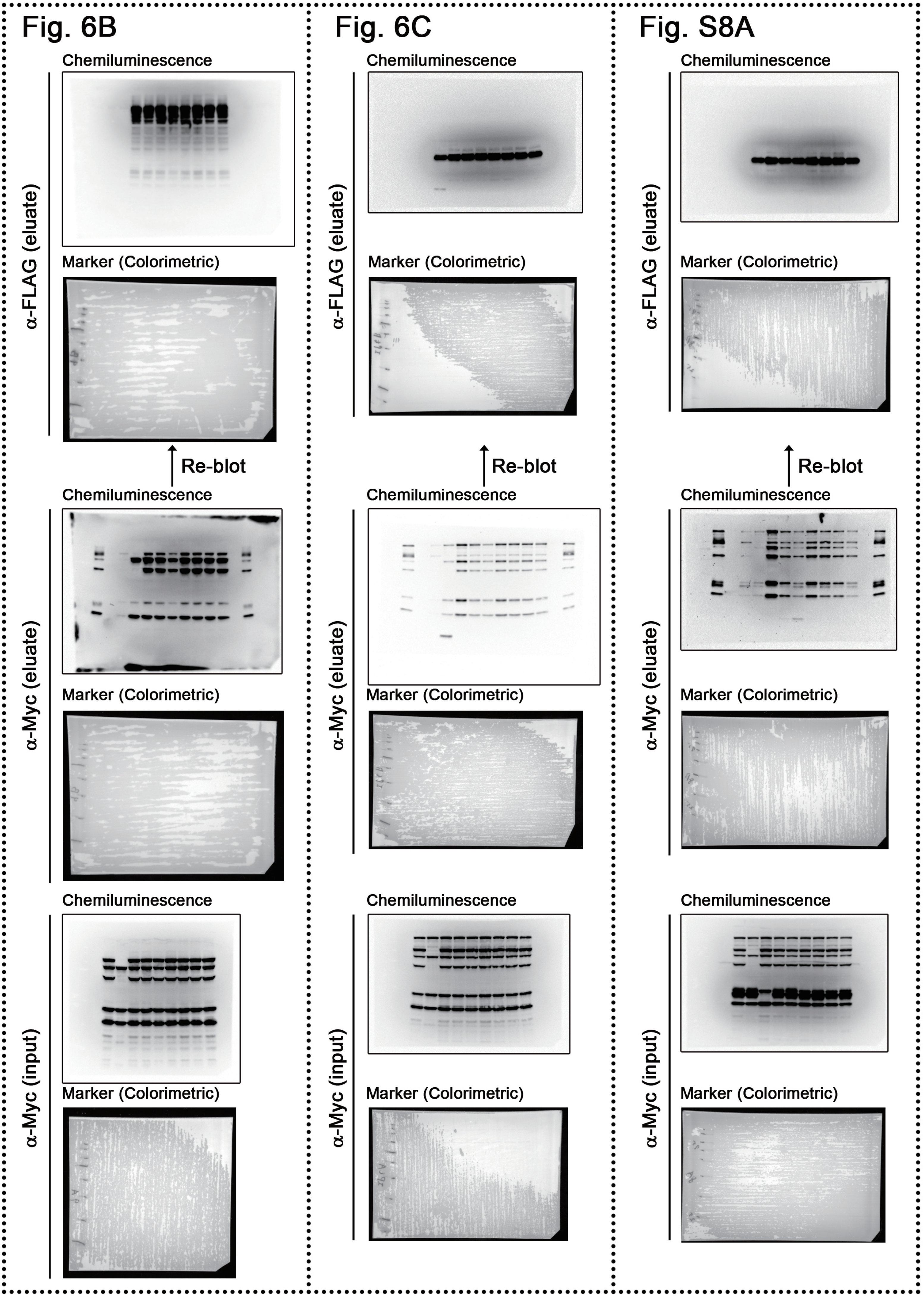

Fig. 7B

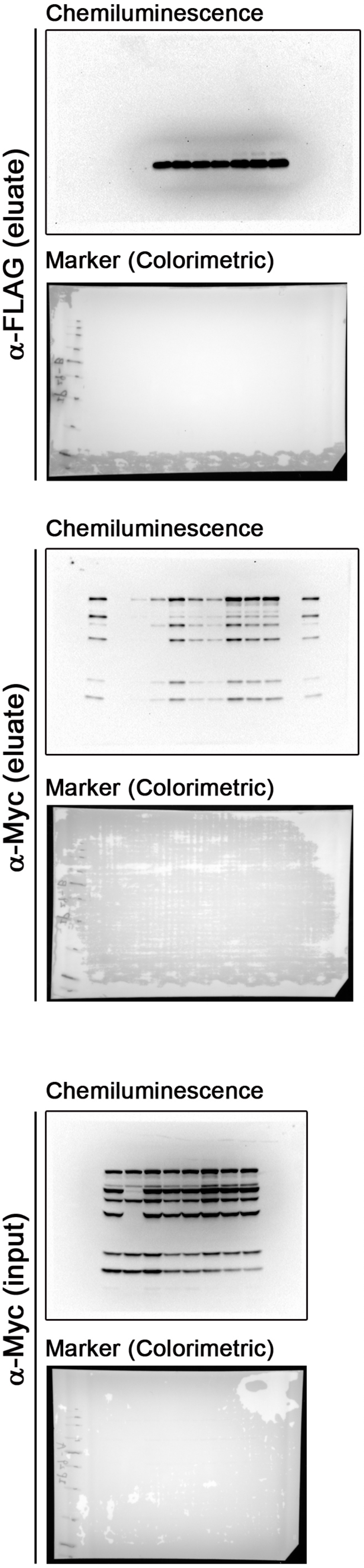

Fig. 7C

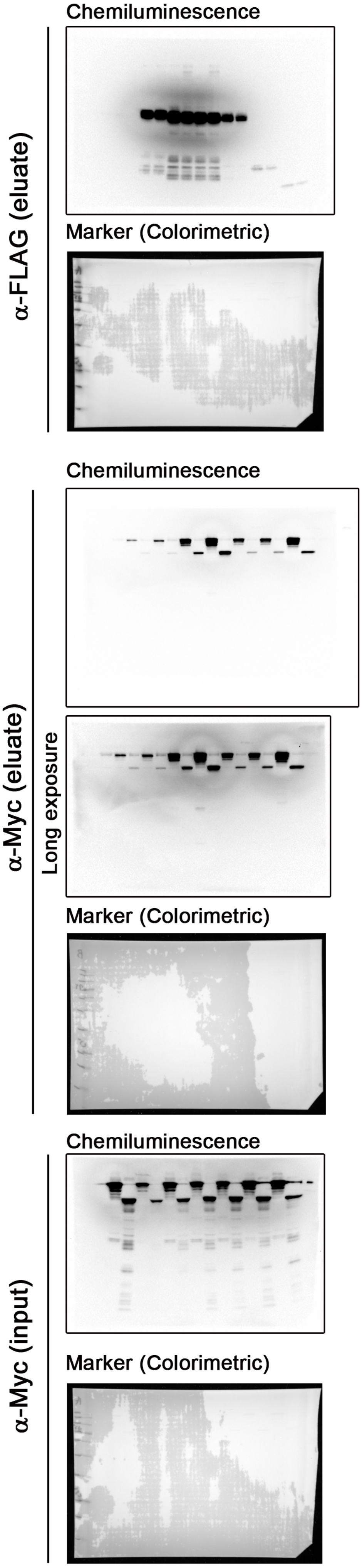

Fig. S9A

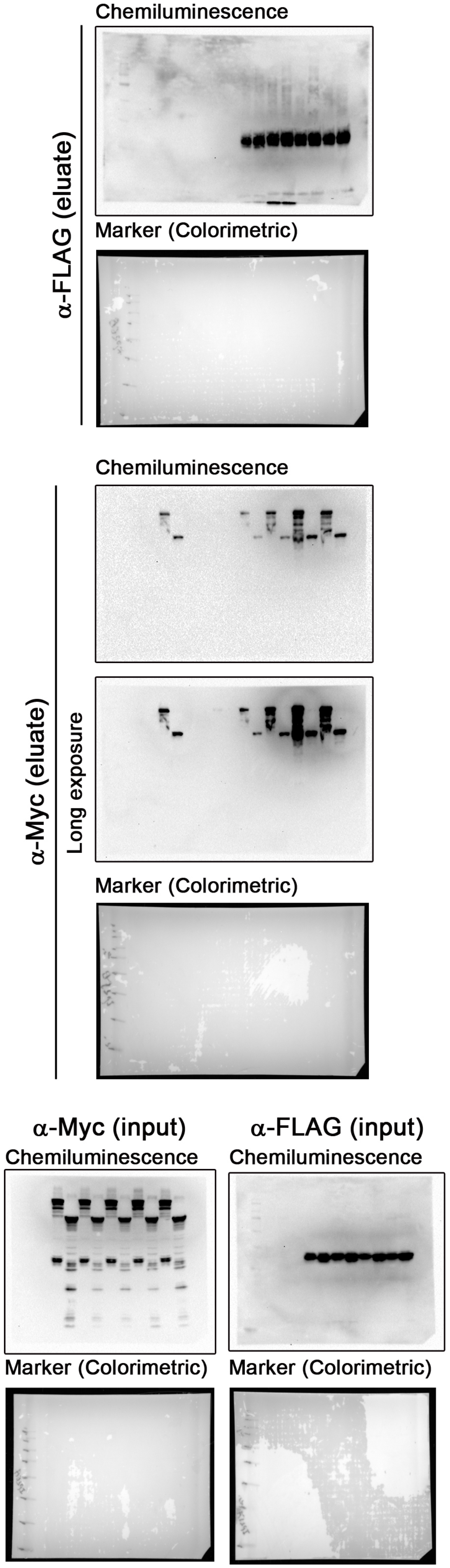

Fig. S7A

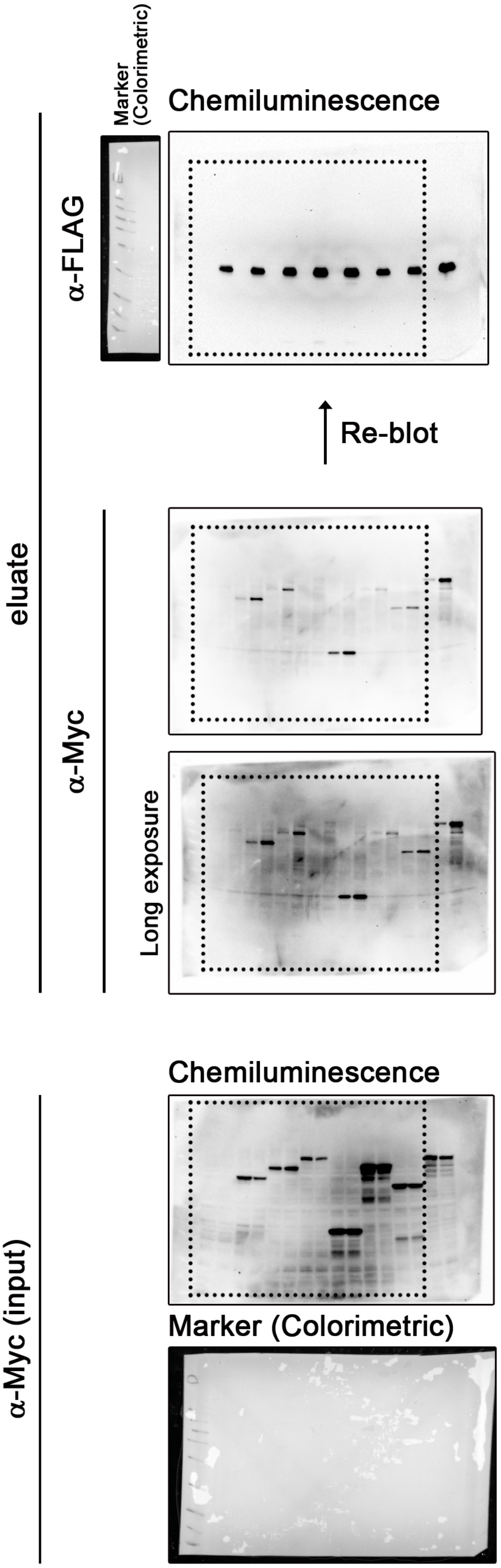

Fig. S7B

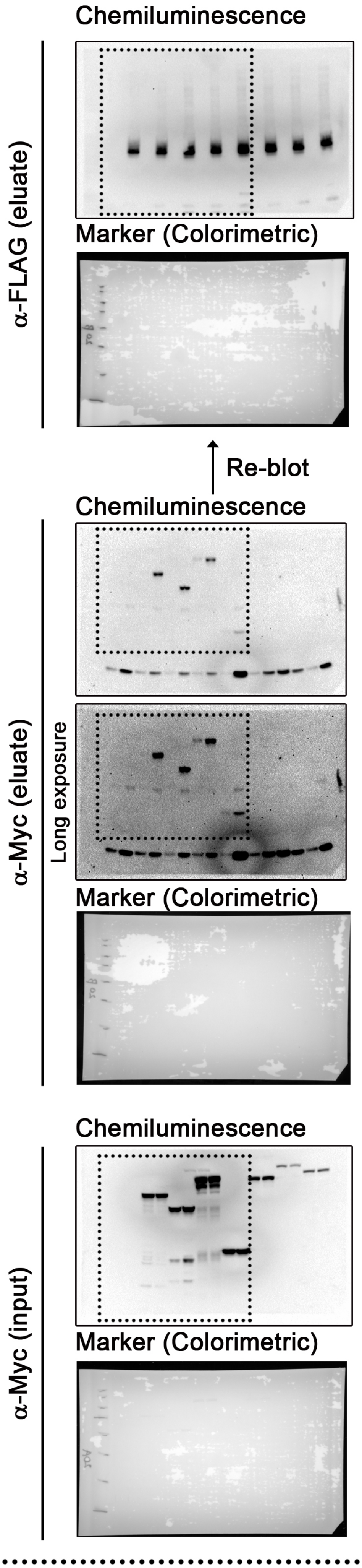

Immunoblot images in Fig. 8

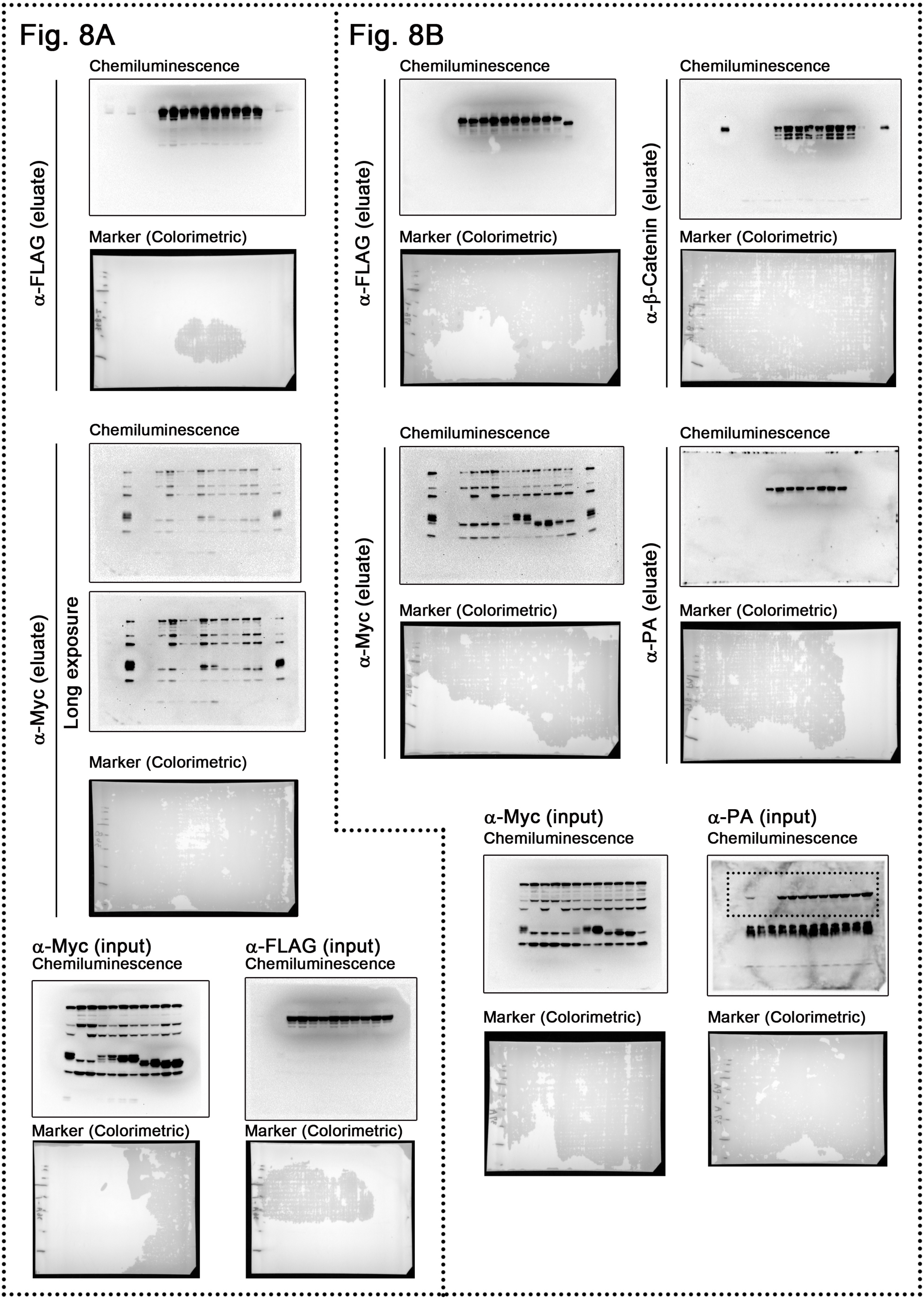

Immunoblot images in Fig. S10

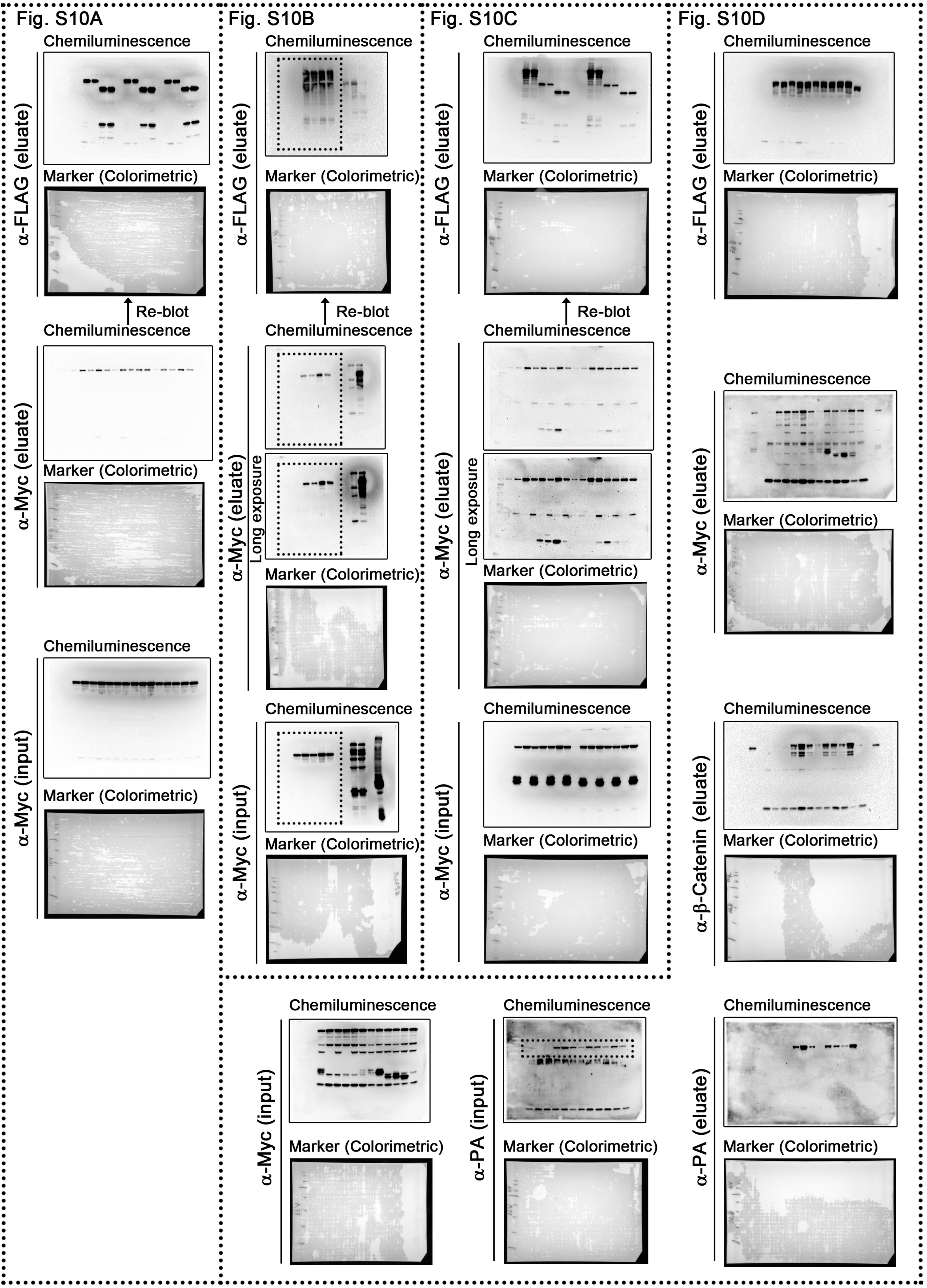

Supplement: Supplementary file 3 — Original gel images [file 41419_2025_7737_MOESM3_ESM.pdf]
